# Ocular markers of microangiopathy and their possible association with cardiovascular risk in patients with systemic inflammatory rheumatic diseases: a systematic review

**DOI:** 10.3389/fimmu.2025.1543157

**Published:** 2025-04-15

**Authors:** Bengta Sturm, Anna-Lena Zang, Julia Stingl, Rebecca Hasseli-Fräbel, Antonis Fanouriakis, Andreas Schwarting, Christian Geber, Julia Weinmann-Menke, Mohammed Alhaddad, Konstantinos Triantafyllias

**Affiliations:** ^1^ Department of Internal Medicine I, Division of Rheumatology and Clinical Immunology, Johannes Gutenberg University Medical Center, Mainz, Germany; ^2^ Department of Ophthalmology, Johannes Gutenberg University Medical Center, Mainz, Germany; ^3^ Center for Translational Rheumatology und Immunology, Institute of Musculoskeletal Medicine (IMM), University of Münster, Münster, Germany; ^4^ Rheumatology and Immunology Department, Attikon University Hospital, National and Kapodistrian Universitiy of Athens, Athens, Greece; ^5^ Department of Rheumatology, Acute Rheumatology Center Rhineland-Palatinate, Bad Kreuznach, Germany; ^6^ DRK Schmerz-Zentrum Mainz, Mainz, Germany; ^7^ Division of Rheumatology and Clinical Immunology, Department of Internal Medicine I, Johannes Gutenberg University Medical Centre, Mainz, Germany

**Keywords:** retinal vessel analysis (RVA), optical coherence tomography angiography (OCT-A), spectral domain optical coherence tomography (SD-OCT), retrobulbar color Doppler, microangiopathy, systemic lupus erythematosus (SLE), rheumatoid arthritis (RA), systemic sclerosis (SSc)

## Abstract

Individuals with autoimmune rheumatic diseases (ARDs) are at a higher cardiovascular (CV) risk due to systemic inflammation, which contributes to endothelial dysfunction, atherosclerosis, and structural changes in the vessel walls. Along with traditional CV risk factors like dyslipidaemia, arterial hypertension, obesity, and impaired glucose metabolism, these patients have a severe prognosis with higher CV morbidity and mortality rates. To date, there is limited data on the optimal CV screening methods for individuals with ARDs, as conventional risk algorithms may underestimate the influence of chronic inflammation. In comparison to macrovascular assessment methods, such as carotid-femoral pulse wave velocity and carotid sonography, microvascular changes, which may precede macrovascular disease, have been less investigated. The ocular microvasculature reflects systemic vascular health and can reveal early signs of CV disease. Changes in retinal vessels have been linked to an increased long-term risk of CV mortality and ischemic stroke in longitudinal studies of the general population, such as the large Atherosclerosis Risk in Communities (ARIC) study. Additionally, various cross-sectional and follow-up studies in patients with ARDs have demonstrated associations between ocular vessel changes, traditional CV risk scores, and disease-related characteristics, suggesting a potential role for ocular assessments in CV risk screening. In this review work, research from 26 studies retrieved from the PubMed and Web of Science databases has been highlighted. Herein, we evaluate the techniques of retinal vessel analysis (RVA), optical coherence tomography angiography (OCT-A), spectral domain-OCT (SD-OCT), and retrobulbar color Doppler. Specifically, we examine the available data on their associations with key CV risk factors, systemic inflammation, surrogate CV markers, and traditional CV risk scores. Furthermore, we discuss their potential diagnostic value in both ARDs and the general population. Despite current limitations, such as small sample sizes and methodological heterogeneity, initial findings suggest that these techniques may provide valuable insights into microangiopathy and CV risk. Future research should focus on larger, well-designed longitudinal studies to establish their prognostic value and potential integration into clinical practice.

## 1 Introduction

Rheumatic diseases originate from autoimmune mechanisms, whereby the immune system mistakenly attacks the body’s own tissues (1). These diseases are characterized by chronic inflammation affecting various organs, occurring as a result of a genetic predisposition and environmental triggers (1). Systemic inflammatory diseases, such as arthritides and connective tissue disorders, are associated with an increased risk of comorbidities including CV events (e.g. stroke, coronary artery occlusion myocardial infarction) (2–7). Additionally, these diseases are linked to increased morbidity and mortality rates (8–13). Patients with systemic lupus erythematosus (SLE) face a twofold increased risk of CV mortality, while those with rheumatoid arthritis (RA) have a 1.5-fold higher risk of CV events (14). Chronic systemic inflammation underlies endothelial dysfunction and accelerated atherosclerosis (15, 16).

Additional factors contributing to the higher CV risk in rheumatic diseases include anti-inflammatory therapies like glucocorticoids, which may cause dyslipidemia, hypertension, and a diabetic metabolic state (17). Research in SLE and other ARDs suggests that ongoing systemic inflammatory may accelerate atherosclerosis and increase CV risk, even during disease remission. Evidence indicates that CV events occur up to two years prior to the formal SLE diagnosis (15). These findings highlight the need for surrogate markers to identify patients at risk, enabling early intervention and improved outcomes.

### 1.1 Traditional risk scores

Many traditional CV risk assessment scores, such as Systematic Coronary Risk Evaluation (SCORE/SCORE2), the Prospective Cardiovascular Münster Study (PROCAM) score, and the Framingham Risk Score (FRS) (18–20) were developed for the general population. They depend on major modifiable CV-risk factors (18, 21, 22). SCORE/SCORE2 estimates 10-year CV risk in Europeans aged 40 to 69 without a history of CVD or diabetes, with SCORE2-OP involving those aged 70 and above. It considers blood lipid values, systolic blood pressure, age, sex, and smoking (23, 24). The European Alliance of Associations for Rheumatology (EULAR) recommends SCORE2 for assessing CV risk in patients with RA, axial spondylarthritis (AS), and psoriatic arthritis (PsA), using a 1.5 multiplication factor to adjust for RA (25, 26).

The PROCAM score, which is used in Germany, focuses on coronary heart disease (CHD) risk and includes lipid values, systolic blood pressure, smoking, family history, and diabetes (27). The FRS estimates 10-year CHD risk based on variables like blood pressure and cholesterol (28, 29). To improve screening for connective tissue diseases, additional factors like mental illness, SLE, RA, chronic kidney disease, atrial fibrillation and family history of CVD were included in the modified FRS and SCORE and new scores such as QRISK2 and QRISK3 were introduced (30, 31). However, evidence is limited on whether scores developed for the general population could apply accurately to rheumatic patients, and the efficacy of these adjusted scores in predicting CV risk remains inconclusive (25, 26, 32, 33). This highlights the need for further surrogate markers for a more precise CV risk assessment (34).

### 1.2 Surrogate CV markers

Most studies on surrogate CV markers focus on large vessels, including arterial stiffness, carotid sonography, Ankle-Brachial-Index (ABI)-Doppler and other macroangiopathy indicators (35–41). Our research group and others have examined carotid-femoral pulse-wave velocity (cfPWV) and carotid sonography in ARDs, such as RA (42), PsA (43), mixed connective tissue disease (44), SLE (45), antisynthetase syndrome (33), idiopathic inflammatory myopathies (46) and fibromyalgia (47).

**Figure 1:**
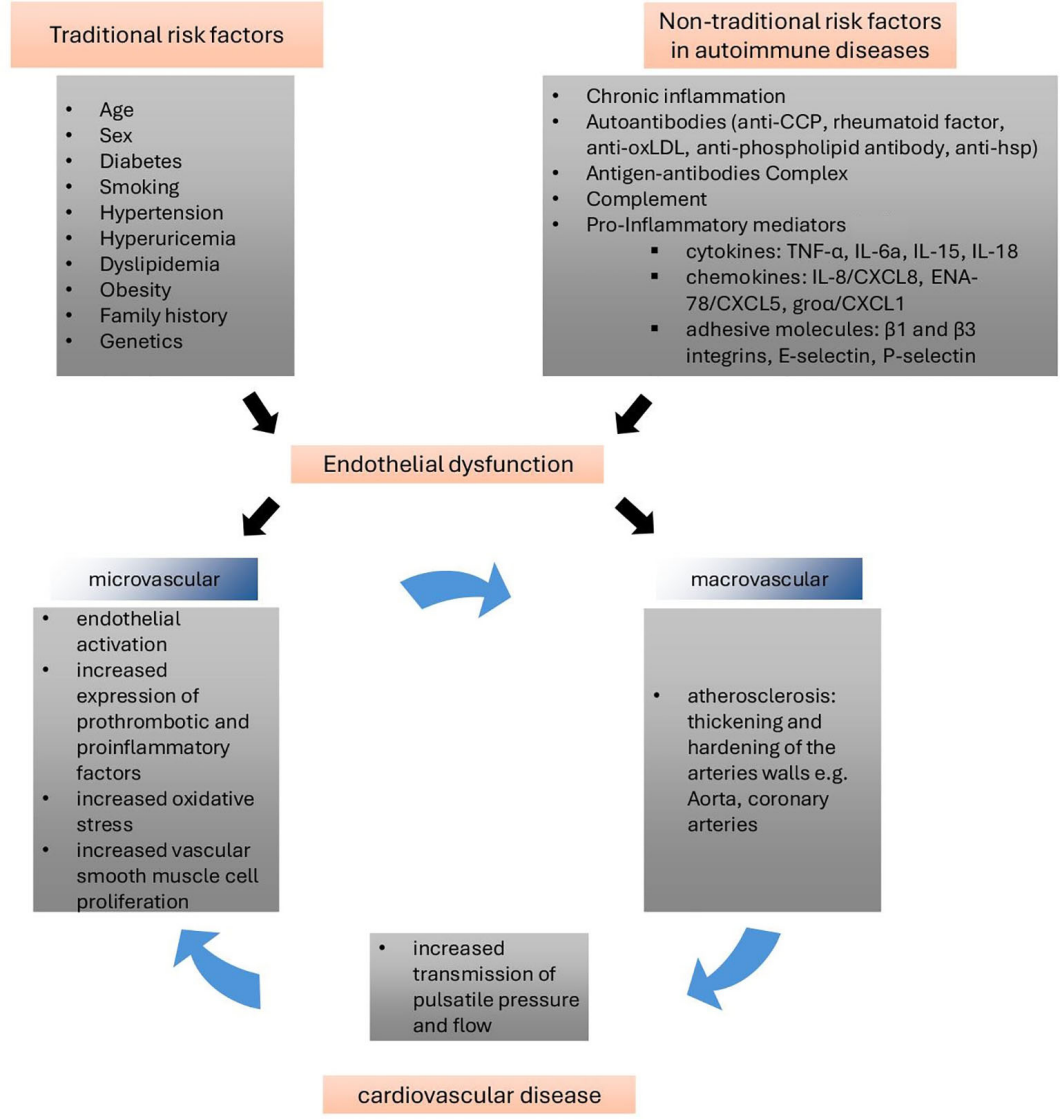
Vicious circle of endothelial dysfunction in ARDs (48, 49). Endothelial dysfunction, driven by traditional and non-traditional risk factors, plays a pivotal role in macro- and microvascular changes, contributing to significant cardiovascular morbidity and mortality. From a pathophysiological standpoint, microvascular dysfunction may serve as a precursor to large artery remodeling, as chronic inflammation could accelerate the atherosclerotic process. Conversely, the stiffening of large vessels amplifies pulsatile pressure transmission to the microvasculature, causing damage to microvascular beds and end organs, thus creating a vicious cycle between small and large vessels. Anti-CCP: anti-cyclic citrullinated peptide; anti-oxLDL: anti-oxidized low-density lipoprotein; TNF-α: tumor necrosis factor-alpha; IL: interleukin; ENA-78: epithelial neutrophil-activating protein-78; groα: growth-regulated oncogene-alpha. (adapted from 48).

Conversely, data concerning arterial alterations of the microvasculature are scarce. Nevertheless, the link between microvascular and macrovascular changes has been established, with endothelial dysfunction as a critical initiating factor in a deleterious cycle (48–51) (**Figure 1**). In a recent review by Hysa et al. (52), the authors discuss how ARDs lead to ocular microvascular damage through immune complex formation, complement activation, and antibody-mediated endothelial injury. This endothelial dysfunction in ocular vessels reflects broader vascular involvement, suggesting that retinal microvascular changes could serve as early indicators of systemic vascular damage.

Importantly, arterial stiffness in the microvasculature of target organs can lead to CV complications, such as isolated systolic hypertension and increased pulse pressure (53, 54). Retinal vessels are particularly suitable for assessing microvascular health, and the retinal vascular phenotype has been shown to be predictive of CV risk (55). Methods like static and dynamic retinal vessel analysis (SRVA, DRVA) have been extensively studied in the general population (55). Additionally, advanced ocular examinations, such as optical coherence tomography with angiography or analysis with a spectrometer (56–58) and doppler ultrasound of ocular vessels have been employed to determine retinal blood flow and vascular resistance in cross-sectional studies (59–61).

Interestingly, data on ocular markers of angiopathy and CV risk in rheumatic diseases are limited and have not yet been collectively presented. This review provides an overview of the existing studies on retinal vessel examination methods and their potential value in assessing CV risk in ARDs.

## 2 Pathophysiology

**Figure 2:**
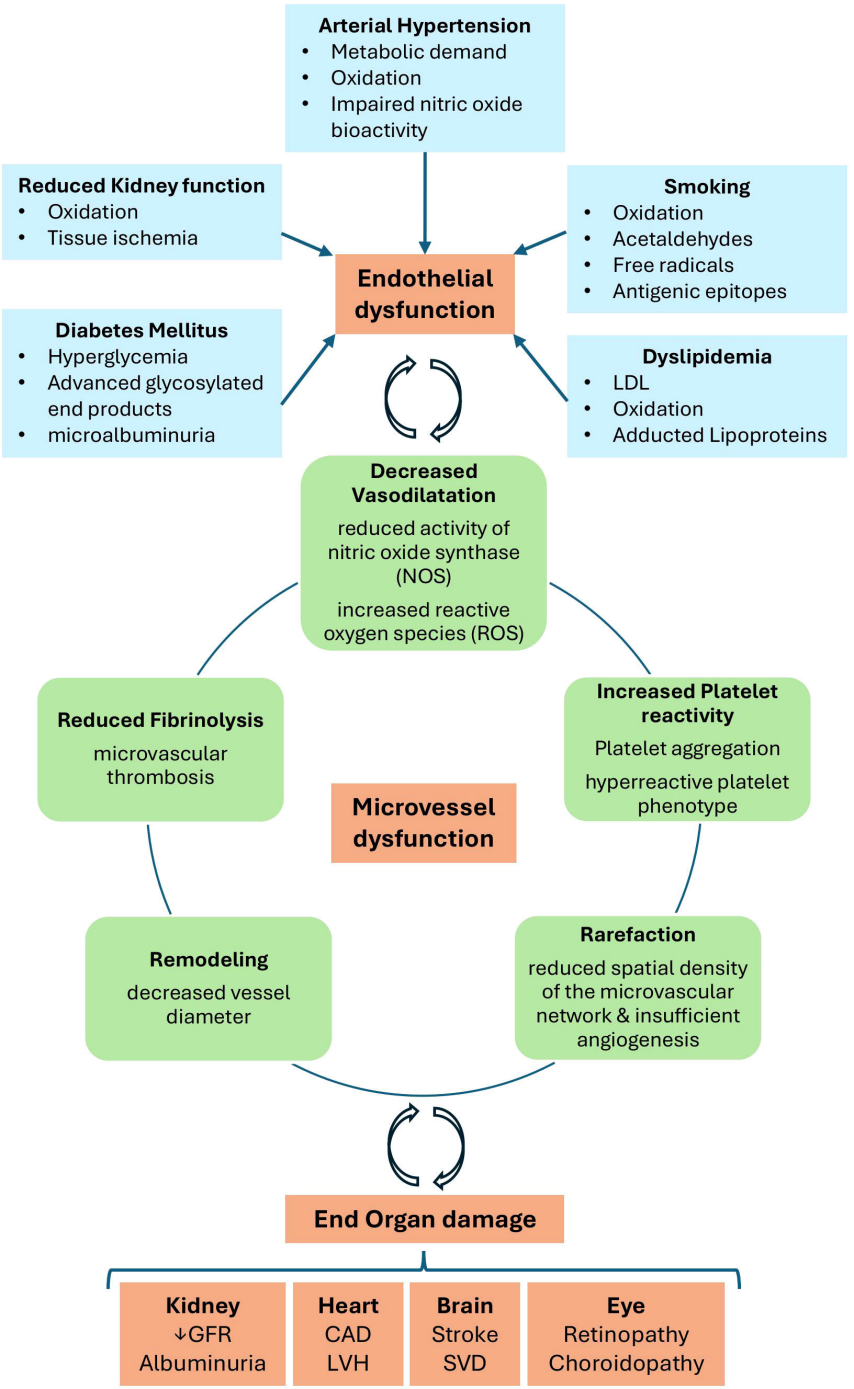
Initiation and consequences of microvascular disease (69, 70). Several factors including smoking, dyslipidemia, diabetes mellitus, arterial hypertension and reduced kidney function, contribute through multiple mechanisms to microvascular damage, ultimately leading to the development and progression of end-organ dysfunction. CAD, coronary artery disease; GFR, glomerular filtration rate; LVH, left ventricular hypertrophy; SVD, small vessel disease (adapted from Farrah et al., 2020).

The microvasculature encompasses the smallest components of the circulatory system, consisting of blood vessels with diameters less than 300 μm (62). It comprises arterioles, capillaries, and venules, which facilitate the delivery of oxygen and nutrients to tissues while enabling the removal of metabolic waste products. These vessels allow essential exchange between the bloodstream and surrounding cells, crucial for maintaining tissue and organ functions. Moreover, they are involved in the regulation of immune responses within the body (63). Endothelial cells, in conjunction with the smooth muscle cells that coat the vessels, are responsible for maintaining vessel tone and regulating blood flow. Their function is highly dependent on oxygen (62–64). Should vessel alterations occur, in terms of vascular density or morphology, the viability of the surrounding tissue and organs can be jeopardized (65, 66). Endothelial dysfunction, characterized by the production of reactive oxygen species (ROS) and reduced availability of nitric oxide (NO), is a pivotal mediator influenced by intrinsic factors within endothelial cells (63, 67). It is postulated that these ROS, which are triggered by CV risk factors such as smoking, hypertension, and hyperglycemia, initiate a cascade of angiopathy-associated events (67). Initially, these processes contribute to endothelial dysfunction, which subsequently leads to arteriolosclerosis and atherosclerosis (67). The impairment of the small vessels due to structural changes, functional dilatation, and platelet activation or thrombotic microangiopathies leads to an increase in vascular resistance, and thus to reduced perfusion of end-organs, such as the eyes, kidneys, lungs, brain and heart (67–70) (**Figure 2**).

Embryologically, the retina originates from diencephalon, as does the optic nerve. Consequently, there are similarities between the central nervous system and the cerebrovascular bed (55). Similar to the blood-brain barrier (BBB), there is also a blood-retina barrier, which is intended to protect the vulnerable retinal tissue from potential insults (e.g. immune cells, larger molecules). However, the permeability of the blood-retina barrier is greater than that of the BBB, which is why the retina is more exposed to oxidative stress (55, 71). This explains why the retina is susceptible to systemic CV risks, as ROS play a crucial role in the pathogenesis of atherosclerosis and manifestation of CVD (72, 73). One of the earliest signs of arterial hypertension are microvascular changes, such as an impaired vasomotor function, which can lead to enhanced vasoconstriction or reduced vasodilation. Additionally, there may be anatomic alterations, such as tortuosity, and a rarefaction of arterioles or capillaries. The narrowing of retinal arterioles is indicative of an elevated systemic vascular tone (74). A substantial body of evidence has demonstrated a robust and independent correlation between blood pressure and arteriolar narrowing of retinal vessels (75–77). Therefore, the retinal vessels represent an intriguing interface, wherein the association of alterations in retinal microvascular endothelial dysfunction with systemic CV risk factors can be estimated (78, 79).

## 3 Methods

**Figure 3:**
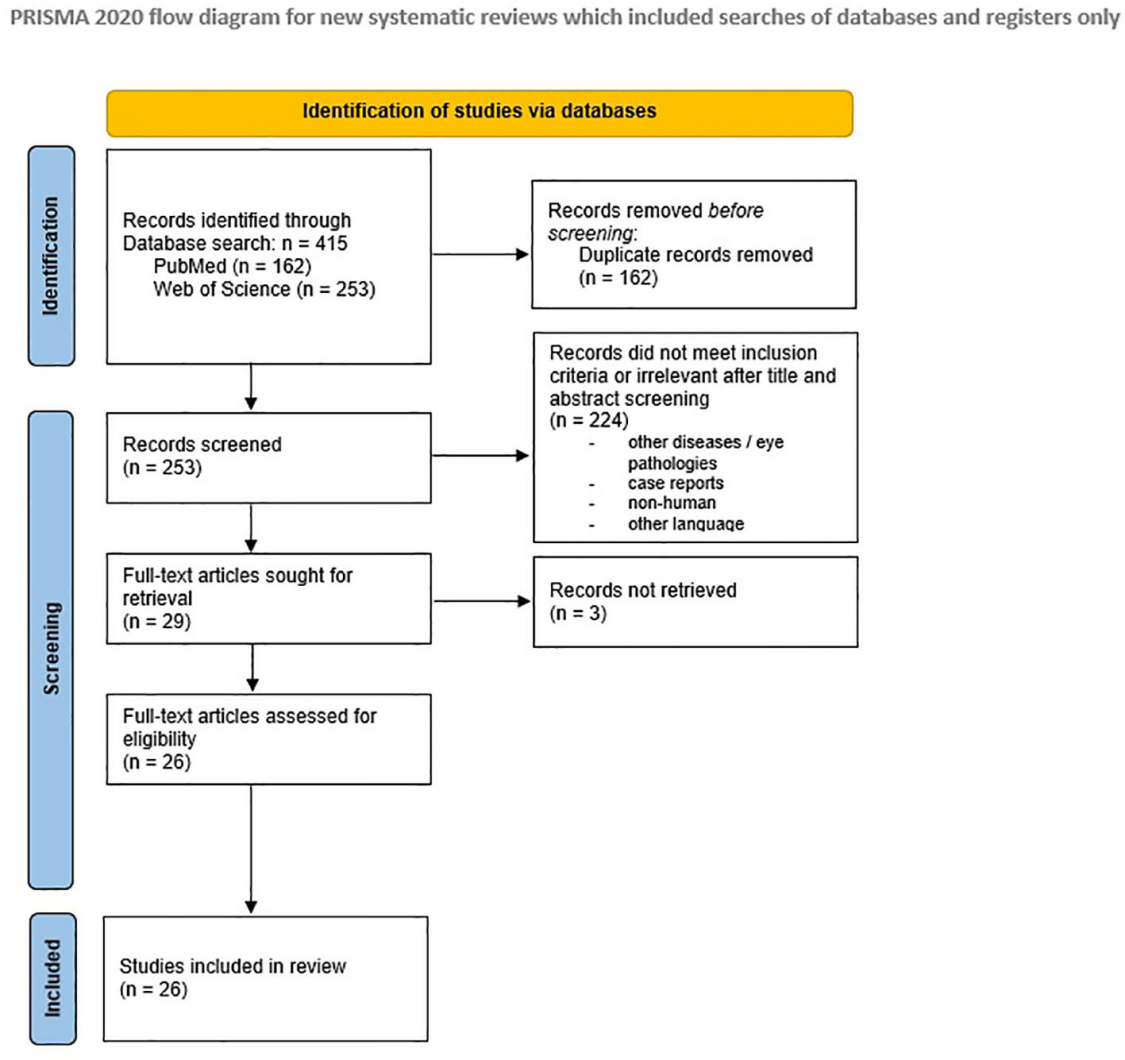
PRISMA 2020 flow diagram (125).

We employed a search strategy to identify relevant literature (**Figure 3**). Given the limited data available on ARDs, we included various study designs, such as cross-sectional and longitudinal studies, as well as case-cohort and registry-based analyses. Additionally, relevant long-term follow-up studies and meta-analyses from the general population were considered. We focused on rheumatic diseases with a well-documented and prominent microangiopathy, such as systemic sclerosis (SSc) and systemic lupus erythematosus (SLE). Moreover, we included the most common inflammatory arthritis, rheumatoid arthritis (RA), due to its high prevalence and established association with cardiovascular (CV) risk. Our selection was guided by both the availability of relevant studies and the strength of the association between these diseases and vascular changes.

Following the PICO framework, we considered SLE, RA and SSc, as the patient/population (P), retinal vessel examination as the intervention (I), a healthy population as the control group/comparison (C), and microangiopathy or CV risk as the outcome (O). The search was performed on PubMed and Web of Science from 14 November 2023 to 21 June 2024 with the following inclusion criteria: all relevant studies, written in English language, published after 1990. Case reports and papers with only published abstracts were excluded. We first used the key words “retinal vessel”, “cardiovascular risk”, as well as (“rheumatoid arthritis” OR “systemic lupus erythematosus” OR “systemic sclerosis”) linked with the Boolean operator AND. This yielded a total of 8 results on PubMed and 11 on Web of Science. Consequently, the search terms were modified by employing truncation in the keyword strategy, in order to expand the search results. The combination of the keywords “microvasc*” and “cardiovascular risk” in combination with “rheumatoid arthritis” OR “systemic lupus erythematosus” OR “systemic sclerosis” produced a total of 130 results on PubMed and 267 on Web of Science. Restricting the research to results published in the last two decades and including only full-text publications in English yielded 123 publications on PubMed and 218 on Web of Science, respectively. Subsequently, the keyword combination was modified, in order to retrieve further useful results. This involved exchanging “microvasc*” with “ocular” or “retinal”, resulting in additional 74 results. Following manual exclusion of duplicate records and unretrieved items, a total of 253 records were subjected to further analysis. After excluding results that did not meet inclusion criteria or were irrelevant, 29 full-text articles were identified, of which 3 could not be retrieved. In total, 26 articles and studies were included in this review.

## 4 Methods of assessment of ocular microangiopathy markers

### 4.1 Retinal vessel analysis (RVA)

#### 4.1.1 Definition of RVA

**Figure 4:**
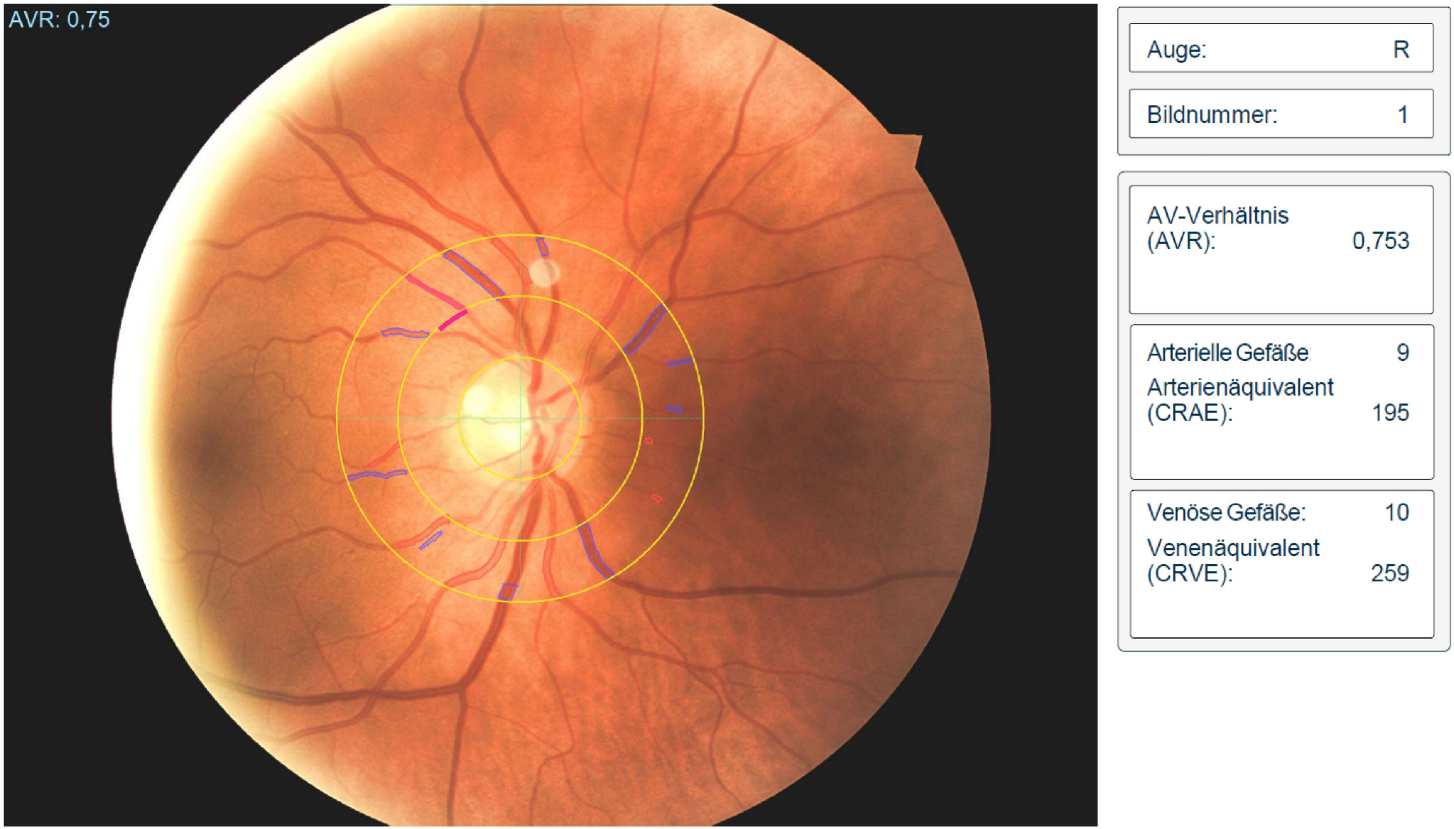
Retinal vessel analysis of a patient with systemic lupus erythematosus (AVR<0.78 associated with higher CV-Risk) (77).

RVA is a non-invasive and non-mydriatic assessment method that employs ocular fundus vessel photography to calculate the diameters of retinal arterioles and venules. It involves the examination and measurement of the smallest blood vessels in the retina. Using a dedicated camera, RVA can be conducted either statically (capturing a single picture) (**Figure 4**) or dynamically (taking a series of pictures, after stimulating the regulation mechanism with a stimulus such as flicker light). Subsequently, the generated image is evaluated using specialized software, such as the “Vesselmap Analysis” (62).

The diameters of the retinal vessels are automatically measured, and vascular parameters are typically calculated using the formulas described during a large long-term epidemiologic study aimed at investigating the causes and risk factors for CVD, the ARIC study (80). This study included approximately 15,000 participants from four communities in the United States and documented various traditional CV risk factors, including high blood pressure, diabetes, and lifestyle habits to evaluate the diagnostic value of RVA in the assessment of the risk for myocardial infarction, stroke, and other CVD (80). The study found an important association between mean arterial pressure (MAP) with AVR which decreased after every 10 mmHg increase in MAP. The diameters of the retinal vessels were calculated in accordance with the dimensions and hemodynamics of the retinal microvasculature, employing a formula to quantify vessel narrowing as an arteriolar-venular ratio (AVR) (80, 81). To facilitate comparison of arteries across different eyes, the width of the central retinal artery (CRA), considered as the main trunk, was calculated based on measurements of all retinal arteries. Similar measures were performed for the venous caliber (81). These parameters are commonly expressed as central retinal venular equivalent (CRVE), central retinal arteriolar equivalent (CRAE), and AVR. The ratio of CRAE and CRVE is used to calculate the AVR and these parameters are used to quantify the average of the retinal vessels. The equivalents represent the central arterial inflow and venous outflow of the retina. The AVR serves as a marker reflecting the regulatory state of the retinal microcirculation (80, 81).

Imaging of constricted retinal vessels can provide information about ocular vascular impairment, thus serving as a surrogate marker for potential subclinical arteriosclerosis in the small vessels (67, 80). Software used for analysis is presented in (**Table 1**).

**Table 1:** Methods of ocular microangiopathy assessment.

	Software	Region of development
Retinal vessel analysis	Retinal Analysis (RA)	University of Wisconsin, Madison, WI
	Integrative Vessel Analysis (IVAN)	University of Wisconsin, Madison, WI
	Singapore “I” Vessel Assessment (SIVA)	National University of Singapore
	Vascular Assessment and Measurement Platform for Images of the Retina (VAMPIRE)	International collaborative project
Optical coherence tomography-angiography	AngioVue optical coherence tomography angiography	Optovue Inc., Fremont, CA, USA
	Cirrus HD-OCT Review Software	Carl Zeiss Meditec. Inc., Jena, Germany
	IMAGEnet	Topcon, Japan
	OCTAVA	National Institutes of Health in Bethesda, Maryland, USA
	ImageJ	National Institutes of Health in Bethesda, Maryland, USA
Spectral Domain Optical Coherence Tomography	Heidelberg Spectralis	Heidelberg Engineering, Heidelberg, Germany
	Cirrus HD-OCT	Carl Zeiss Meditec Inc, Dublin, CA

#### 4.1.2 Evidence from the general population

Hubbard et al. (80), in the population-based, cross-sectional ARIC cohort study, first described a significant correlation between retinal microvascular changes and MAP. Among 9,040 patients, focal arteriolar narrowing (OR 2.95, 95% CI 1.87–2.14) and arteriovenous nicking (OR 1.25, 95% CI 1.16–1.34) were observed in 7% and 6% of the cases, respectively. Patients with arteriolar stenosis had an 8 mmHg higher MAP, and patients with nicking showed a 3 mmHg increase (both p < 0.0001). During a mean follow-up of 16 years form an ARIC cohort, Seidelmann et al. (77) showed narrower retinal arteries (HR 1.06, 95% CI 1.01–1.11) and wider venules (HR 1.13, 95% CI 1.08–1.18) correlated with higher long-term risk of death and stroke. In a 15-year follow-up from a subgroup of the Inter99 study (study of CV and metabolic characteristics and lifestyle), Drobnjak et al. (82) found narrower CRAE associated with higher systolic blood pressure (p < 0.001), age (p = 0.002), and higher HDL (p = 0.003), while wider CRAE and CRVE were associated to smoking (both p < 0.001). Wong et al. (83) also confirmed in the Multi-Ethnic Study of Atherosclerosis study (MESA), within 5979 people between the ages of 45 and 84 residing in six US communities, associations between wider retinal vessels, smoking and diabetes. In a subgroup of the MESA study, Kawasaki et al. found narrower CRAE (OR 1.34) and wider CRVE (OR 1.18) were related to hypertension development after approximately 3 years of follow-up (84). Cheung et al. (74) found low HDL correlated with venous tortuosity, while high blood pressure, BMI, and age were associated with arterial tortuosity.

To summarize, RVA has been shown to correlate with CV events (e.g. stroke), CV mortality, and CV risk factors like hypertension, smoking, diabetes, age, BMI and blood lipids in the general population. It is therefore considered a non-invasive, easily reproducible method for assessing cardiovascular risk and potential disease progression.

#### 4.1.3 Evidence in patients with autoimmune rheumatic diseases

Data on the value of RVA in rheumatic diseases are relatively scarce; most studies have employed a cross-sectional design and small sample sizes.

Lee et al. examined 50 SLE patients and an equal number of control subjects, matched for age and other CV risk factors, including BMI, arterial pressure, and cholesterol levels. The researchers observed narrower CRAE and CRVE and lower AVR in the patient group compared to controls. However, these findings did not reach statistical significance (CRAE: 89.7 ± 14.5 vs. 102.2 ± 11.3 μm, p=0.154; CRVE: 127.7 ± 14.8 144.1 ± 14.2 μm, p=0.609; AVR 0.69 ± 0.54 vs. 0.71 ± 0.66 p=0.223) (85).

Regarding association with activity indices, Babaoğlu et al. examined 47 RA patients, 32 SLE patients and 45 healthy controls by RVA after fundus photography (86). The authors found that elevated CRVE was associated with higher DAS28 values, underscoring an association between disease activity and retinal microangiopathy in the context of the disease. This can be an indicator of an association between disease activity and increased CV risk. Interestingly, this association was independent of CRP levels, indicating that angiopathy may also be found in the absence of laboratory inflammatory activity.

Larger meta-analyses, such as that of Liu et al., have sought to describe the relationship between inflammatory processes (primarily reflected in elevated CRP levels) and microcirculatory changes in the retina (87). This meta-analysis included 36 studies and tried to investigate whether there was an association between inflammation and altered retinal microvascular parameters in the general healthy population compared to patients with inflammatory diseases. The analysis included cohorts of patients with ARDs, such as RA and SSc, as well as CVD, high arterial pressure, obesity, type 2 diabetes mellitus, and acquired immunodeficiency syndrome (AIDS). A link between CRP and venous caliber was described in just over 20 studies. In summary, no evidence could be shown for the connection between retinal arteriolar caliber and CRP. However, there was a correlation between venous caliber and CRP (r = 0.09, 95% CI 0.05 to 0.12).

Interestingly, in patients with ARDs an association between elevated CRP and retinal parameters was only demonstrated in one of the two included RA studies. In this study, it was reported that CRAE was inversely associated with CRP (r = −0.293, p = 0.007). Moreover, retinal venular width correlated weakly with CRP levels (r = 0.218, p = 0.048) (88).

In another study, 26 patients with RA were examined using static RVA, alongside 13 patients with PsA and 12 patients with SpA (89). This patient cohort exhibited impairment of the retinal microvasculature. Specifically, when considering the entire cohort of rheumatic diseases, patients exhibited significantly higher CRVE values compared to a healthy control group, indicating wider venular diameters (Median 221 µm vs. 215 µm, p = 0.01 vs. 215 μm (IQR 196, 223); p = 0.01). Importantly, the patients included in this study were carefully selected to ensure the absence of any classic CV risk factors, such as arterial hypertension. Moreover, the study explored a potential relationship between physical activity (measured by a questionnaire) and vascular changes. Remarkably, just one additional hour of physical exercise per week was found to result in a notable reduction in CRVE, even after adjusting for age, gender, and BMI (CRVE of -0.59 μm; IQR -1.10, -0.08; p = 0.02). Interestingly, these patients did not show any abnormalities in large artery stiffness. Thus, it was postulated that retinal vessel analysis could be a sensitive biomarker to unmask vascular impairments, even in the absence of classic CV risk factors and unremarkable large artery examinations (**Table 2**).

**Table 2:** Selection of studies regarding the value of ocular microangiopathy markers in autoimmune rheumatic diseases.

Method	Authors	Number of patients	Disease	Main results	Statistical significance
RVA	Deiseroth et al. (89)	51	RA & Spondylarthritis	Patients exhibited significantly higher CRVE values compared to healthy controls [median 221 μm (interquartile range (IQR) 211, 231) vs median 215 μm (IQR 196, 223)].	p=0.01
	Anyfanti et al. (88)	87	RA	CRAE and AVR were decreased in patients compared to controls (78.8 ± 8.9 vs 90.2 ± 9.9 μm and 0.69 ± 0.09 vs 0.81 ± 0.09, respectively). CRAE and AVR were inversely associated with CRP (r=−0.293, r=−0.449, respectively). CRVE correlated positively with CRP (r = 0.218).	All, p<0.05
	Babaoğlu et al. (86)	47	RA	Elevated CRVE was associated with higher DAS28 (when DAS28>5.1, CRVE=220.4 μm, and when DAS28≤ 3.2, CRVE=214.4 μm)	p=0.03
	Lee et al. (85)	50	SLE	Narrower CRAE (89.7 ± 14.5 vs. 102.2 ± 11.3 μm) and CRVE (127.7 ± 14.8 vs. 144.1 ± 14.2 μm) and lower AVR (0.69 ± 0.54 vs. 0.71 ± 0.66) in the patient group compared to controls.	p=0.154, p=0.609, p=0.223, respectively
OCT-A	Ayar et al. (93)	106	RA	Retinal capillary plexus density in the macula of patients was lower than in healthy controls (50.99 ± 3.30 vs. 52.08 ± 2.36% in the SCP, 55.65 ± 5.73 vs. 57.53 ± 4.60% in the DCP).	Both, p<0.05
	Lee et al. (92)	12	RA	Macular retina vascular density and total microvascular density were lower in patients compared to control group.	Both, p<0.001
	Arfeen et al. (96)	20	SLE	Reduction of vessel densities in superficial and deep plexus macula regions in patients compared to controls (51.33 ± 3.48 vs. 53.19 ± 1.10% in the SCP, 52.28 ± 6.87 vs. 62.02 ± 1.89% in the DCP).	p= 0.037, p<0.001, respectively
	Ferringo et al. (98)	43	SLE	Reduced vessel density in both superficial and deep retinal vessels of patients compared to healthy controls (50.1 ± 5.6 vs. 53.0 ± 2.3% in the SCP, 55.4 ± 7.0 vs. 58.6 ± 5.4% in the DCP).QRISK3 score and IMT were identified as independent risk factors for changes in the retinal vessels	Both, p<0.05
	Carnevali et al. (99)	20	SSc	Reduced vessel density of the deep capillary plexus in patients compared to controls (47.29 ± 3.49 vs. 50.81 ± 3.71%)	p=0.00
	Hekimsoy et al. (100)	45	SSc	The vessel densities of the superficial and deep capillary plexus were lower in patients compared to controls (49.79 ± 3.47 vs. 51.49 ± 2.89% in the SCP, 51.56 ± 6.52 vs. 52.44 ± 5.43% in the DCP).	Both, p<0.05
SD-OCT	Bao et al. (58)	46	SLE	The density of the retinal capillary plexus was lower in patients than controls (5.3 ± 0.5 vs. 5.8 ± 0.5% in the SCP, 7.0 ± 0.6 vs. 7.4 ± 0.7% in the DCP).	Both, p<0.05
	Pieklarz et al. (113, 114)	33	SSc	The peripapillary choroidal vascularity index was significantly lower in patients compared to controls (64.25 ± 1.94 vs. 65.73 ± 2.12).	p<0.001
Retrobulbar color Doppler	Kal et al. (60)	20	RA	PSV and RI values of patients were higher for the ophthalmic artery and CRA than controls.	All, p<0.05
	Unal et al. (120)	25	RA	Positive correlation was observed between the RI of the ophthalmic artery and DAS 28 (r=0.199).	p=0.02
	Erdogmus et al. (121)	35	RA	Significant differences were observed in PSV, EDV, and RI of the ophthalmic artery and OA, as well as RI of the CRA between the control and patient groups.	All, p<0.05
	Xue et al. (122)	30	SLE	Decreased blood flow velocity in patients along with increased RI and PI in the PCAs and CRA.	p<0.05
	Modrzejewska et al. (123)	43	SLE	Increase in RI in the patient group compared to the controls in all measured arteries.	p<0.01
	Wright et al. (124)	54	SLE	Difference in the morphology of the velocity waveform was observed in patients compared to controls, particularly in the ophthalmic artery and the CRA.	p<0.05

RVA, Retinal Vessel Analysis; CRVE, central retinal venular equivalent; CRAE, central retinal arteriolar equivalent; AVR, arteriolar-to-venular ratio; CRP, C-reactive protein; DAS28, disease activity score 28 joints; OCT-A, Optical Coherence Tomography Angiography; SCP, superficial capillary plexus; DCP, deep capillary plexus; SD-OCT, Spectral Domain Optical Coherence Tomography; PSV, peak systolic flow velocity; RI, resistance index; CRA, central retinal artery; EDV, end diastolic flow velocity; OA, ophthalmic artery; PI, pulsatility index; PCAs, posterior ciliary arteries.

We were not able to find RVA studies specifically in patients with SSc.

To summarize, RVA is a non-invasive method for assessing microvascular health by measuring the diameters of retinal arterioles and venules. It provides important insights into CV risk, as retinal vessel changes, such as narrowed arterioles (expressed by lower values of CRAE) and widened venules (expressed by higher values of CRVE), have been linked to elevated blood pressure, smoking, systemic inflammation, and CV surrogate markers/scores. While RVA has shown significant correlations with traditional CV risk factors in the general population, its application in ARDs remains underexplored. However, available studies suggest that retinal microvascular impairment may serve as an early marker of CV risk in these patients, particularly in relation to disease activity and inflammatory markers.

### 4.2 Optical Coherence Tomography Angiography (OCT-A)

#### 4.2.1 Definition of OCT-A

**Figure 5:**
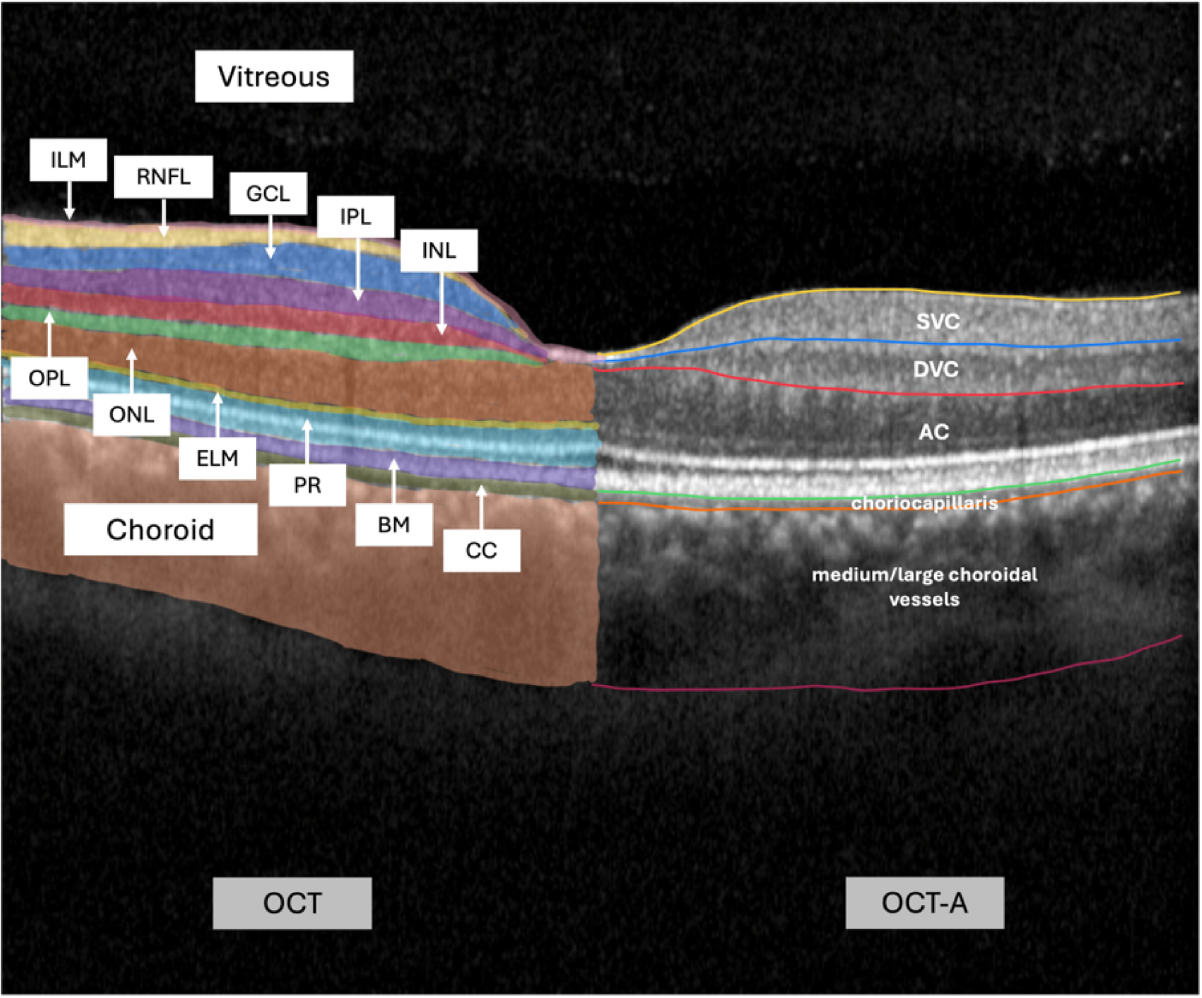
Optical coherence tomography of the macula with labeled retinal layers; left side: layers of structural optical coherence tomography (OCT); right side: layers of optical coherence tomography-angiography (OCT-A). Abbreviations, listed from inner to outer layers, from left section (OCT) to right section (OCT-A): ILM, internal limiting membrane; RNFL, retinal nerve fiber layer; GCL, ganglion cell layer; IPL, internal plexiform layer; INL, inner nuclear layer; OPL, outer plexiform layer; ONL, outer nuclear layer; ELM, external limiting membrane; PR, photoreceptor layers; BM, Bruch membrane; CC, choriocapillaris; SVC, superficial vascular complex/plexus; DVC, deep vascular complex/plexus; AC, avascular complex.

**Figure 6:**
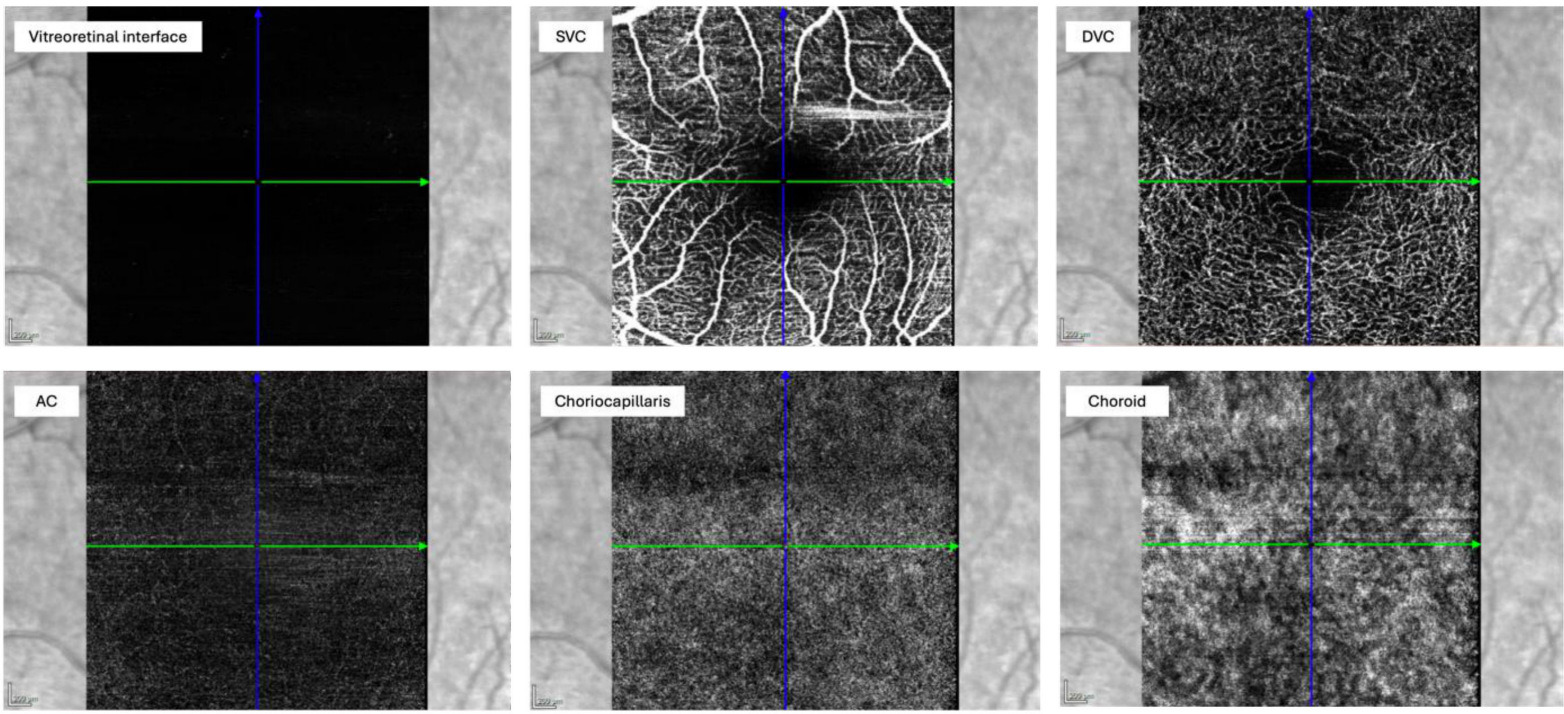
En-face view of the different layers in OCT-A. Abbreviations: SVC, superficial vascular complex/plexus; DVC, deep vascular complex/plexus; AC, avascular complex.

OCT-A is a non-invasive method of blood flow measurement, especially the microcirculation of the posterior segment of the eye, i.e. retina and choroid. This is achieved by visualizing perfused vessels based on the flow registration of their erythrocytes, eliminating the need for injection of fluorescent substances (fluorescein angiography) (56). The perfusion can be visualized in a three-dimensional imaging (**Figures 5**, **6**). The retinal ganglion cells (ganglion cell layer) and their nerve fibers (retinal nerve fiber layer) are supplied by the superficial vascular plexus. The deep vascular plexus is responsible for the inner plexiform, the inner nuclear and the outer plexiform layers, which contain the bipolar, amacrine and horizontal cells, connecting photoreceptors with retinal ganglion cells. The outer retinal layers, anatomically corresponding to the photoreceptors, do not include a vascular network. They are supplied by the choriocapillaris and the medium and large vessels of the choroid. The number of volume scans taken around the fovea varies depending on the method employed (56). Software used for analysis is presented in (**Table 1**).

#### 4.2.2 Evidence from the general population

In a group of patients with diabetic retinopathy, retinal vascular occlusion and age-related macular degeneration, OCT-A demonstrated consistent detection of microaneurysms (capillary saccular outpouchings, early sign for diabetic retinopathy), intraretinal microvascular abnormalities (capillary shunt vessels to provide blood flow in non-perfused areas), non-perfused areas (areas of retinal ischemia due to retinal vessel occlusion, causing neovascular disease), increase of the foveal avascular zone (FAZ) (sign of capillary occlusion around the generally avascular foveal zone) and neovascularization (retinal-choroidal hypoxia leads to vascular ingrowth by release of vascular growth factors) (56).

Sun et al. conducted a case-control study involving 94 eyes from participants with systemic hypertension and 46 eyes from healthy controls. The study found that hypertensive subjects exhibited a significant reduction in macular vessel density in both the superficial [OR 0.02; 95% CI, 0 to 0.64; p = 0.027] and deep venous plexuses [OR 0.03; 95% CI, 0 to 0.41; p = 0.009], along with an enlargement of the deep avascular zone of the fovea compared to controls. These findings suggest that hypertension is associated with reduced retinal vessel density and an increased avascular zone, particularly in the deep venous plexus, as observed using OCT-A. This highlights OCT-A as a promising clinical tool for monitoring hypertensive damage and identifying patients at risk (90).

Importantly, these associations have also been seen in the context of further explorations and ultimately in a meta-analysis evaluating the results of 11 studies on the topic (84). In this meta-analysis it could be shown that the eyes of patients with systemic hypertension have a significantly lower superficial and deep vessel density in the macula compared to healthy control groups. These findings indicate that the OCT measurement approach may offer insights into preclinical microvascular alterations and a connection to an important modifiable CV-risk factor.

To conclude, OCT-A findings in the small retinal vessels could potentially serve as biomarkers for CVD risk stratification related to hypertensive damage in end organs such as the brain, heart, and kidneys (91).

#### 4.2.3 Evidence in patients with autoimmune rheumatic diseases

##### 4.2.3.1 Rheumatoid arthritis

Lee et al. conducted a cross-sectional study in 12 RA patients, demonstrating that macular retina vascular density in various subregional superficial retinal layers, as measured by OCT-A, was lower compared to an age- and sex-matched control group (superficial microvascular density: 1.72 ± 0.07 vs 1.88 ± 0.017 and total microvascular density: 1.71 ± 0.052 vs. 1.78 ± 0.027; both p < 0.001). Moreover, a reduction in vessel density was observed in the deeper retinal layers (92).

Ayar et al. employed a comparable methodology, examining the correlation between OCT-A outcomes and RA disease activity in a cross-sectional study comprising 106 RA patient eyes and 71 healthy control eyes (93). They found that retinal capillary plexus density (CPD) in the macula of RA patients was lower than in healthy controls (50.99 ± 3.30% in RA vs. 52.08 ± 2.36% in HC, p = 0.013). No significant difference was found between active and inactive RA patients (51.01 ± 2.92% in active vs. 50.97 ± 3.73% in inactive RA, p = 0.947).

The clinical relevance of the above observations is not yet clear. The lower vessel density might be a precursor for retinal vasculitis, which however is a rare complication in RA (94). It further may serve as an early biomarker to detect RA-related ocular involvement (92). Still, RA-related complications need to be distinguished from medication side effects, as hydroxychloroquine/chloroquine may cause retinopathy, which may be accompanied by increased FAZ and decreased vessel density in OCT-A (94, 95).

##### 4.2.3.2 Systemic lupus erythematosus

In a cross-sectional observational study, Arfeen et al. evaluated retinal microvascular density in 20 female SLE patients and 20 female control subjects, after excluding patients exhibiting signs of retinopathy (96). The objective was to correlate vascular density with disease activity and damage risk. The findings revealed no statistically significant differences in central foveal thickness (CFT) and FAZ between SLE patients and controls (p > 0.05). Nevertheless, a slight reduction in vessel densities in both superficial and deep plexus macula regions was observed in the SLE patient group (superficial (whole): 51.33 ± 3.48 vs. 53.19 ± 1.10, p = 0.037; deep (whole): 52.28 ± 6.87 vs. 62.02 ± 1.89, p < 0.001). Additionally, an inverse correlation between the SLICC/ACR SDI and vessel density in some macula sectors (p > 0.05) was found. The study concluded that OCT-A can non-invasively assess retinal vessel density, allowing for early detection of altered retinal circulation. The authors suggested that OCT-A could be useful in evaluating disease activity and damage score in SLE patients.

Conigliaro et al. compared a total of 52 eyes of SLE patients to 40 eyes of healthy controls via OCT-A and found reduced retinal microvascular density in SLE patients, particularly in those with kidney involvement (p = 0.02 and p = 0.008) (97). They concluded that vessel density could serve as a quantitative metric for capillary network health, with correlations observed between vessel density and age, best-corrected visual acuity, as well as SLE disease activity and damage accrual. Additionally, the authors proposed that hydroxychloroquine may confer a protective effect on microvascular structures.

In a further cross-sectional study, Ferringo et al. highlighted the increased CV risk in 43 SLE patients, assessed using ACC/AHA and FRS guidelines, and its impact on vascular density measured with OCT-A (98). A negative correlation was observed between deep vessel density and systolic blood pressure (p = 0.011), cIMT (p = 0.027), age (p = 0.001) and QRISK3 Score (p < 0.001). An age- and sex-adjusted multivariate analysis verified that QRISK3 score (p = 0.049) and IMT (p = 0.039) were independent risk factors for reduced retinal vessel density. Additionally, they found higher triglycerides (p = 0.019), FRS (p = 0.008) and reduced vessel density in both superficial (p < 0.001) and deep (p = 0.005) retinal vessels compared to healthy controls (n=34). Importantly, the authors assume that despite low or moderate QRISK3 and FRS levels, SLE patients show striking retinal vascular changes that correlate with subclinical atherosclerosis. These findings suggest that optical OCT-A could play an important role in assessing preclinical cardiovascular involvement in SLE that classic risk scores possibly underestimate.

##### 4.2.3.3 Systemic Sclerosis (SSc)

In a study comprising 20 SSc patients and 20 healthy subjects, Carnevali et al. showed a significantly reduced vessel density of the deep capillary plexus DCP-VD in the SSc group in the whole scan (mean (S.D.) = 47.29 (3.49), p < 0,01) and in the perifoveal (mean (S.D.) = 49.07 (3.02), p < 0,01), superior (mean (S.D.) = 49.41 (3.21), p = 0,02), inferior (mean (S.D.) = 48.72 (3.04), p < 0,01), nasal (mean (S.D.) = 49.46 (3.10), p = 0,01) and temporal (mean (S.D.) = 49.53 (3.33), p = 0,02) regions (99).

In accordance with these findings, Hekimsoy et al. conducted a study involving 45 SSc patients and 45 control subjects (100). The results demonstrated a significant reduction in subfoveal choroidal thickness (SFCT) in SSc patient’s eyes on OCT when compared to healthy subjects (274.47 ± 35.88 vs. 300.95 ± 28.06 p < 0,001). This further supports the effectiveness of OCT-A in visualizing microvascular changes.

In accordance with the aforementioned findings, Mihailovic et al. were able to replicate these results in 22 patients with SSc and 22 healthy controls. The vascular density of the superficial OCT angiogram (OCTA-SCP), and the choriocapillaris (OCTA-CCP) was significantly lower in SSc patients (OCTA-SCP: SSc group: 43.10%, control group: 45.25%, p = 0.036; OCTA-CC: SSc group: 111.07%, control group: 116.96%, p = 0.001). Interestingly, they also reported an association between nailfold capillaroscopy results and vascular density. Their correlation analysis demonstrated a negative correlation between skin score and vascular density of OCTA-SCP (p < 0.05) and a positive correlation between nailfold capillaroscopy and vascular density of OCTA-CC (rho = 0.456, p < 0.05) (101).

A recently published study by Cutolo et al. highlighted the link between peripheral vascular damages (as assessed by nailfold video capillaroscopy (NVC) for morphological microvascular statuses and laser speckle contrast analysis (LASCA) to assess the functional perfusion) and ocular vascular changes detected by OCT-A. An important finding was the direct correlation between the mean capillary count at NVC and the retinal perfusion values of the superficial and deep vascular plexus (SVP and DVP) in OCT-A (r = 0.3, p = 0.01 and r = 0.28, p = 0.01) in 32 SSc patients when compared to 27 sex- and age-matched healthy controls. The mean peripheral perfusion, assessed by LASCA, showed a positive correlation with both the retinal (DVP) and choroidal (choriocapillaris slab, CC) perfusion (r = 0.29 (DVP) and r = 0.28 (CC), both p = 0.01) (102).

A further possible utility of OCT-A seems to be in the distinction between primary Raynaud’s phenomenon (PRP) and SSc, as shown in a cross-sectional study by Erturk et al. (103). A group of 38 SSc patients was included and further divided into very early SSc (VEDOSS), limited cutaneous SSc (lcSSc) and diffuse cutaneous SSC (dcSSC). Herein, it was found that the PRP group exhibited significantly higher whole, parafoveal, and perifoveal SCP and DCP vascular density values compared to the SSc subgroups (all; p < 0.001). In contrast, the lcSSc group had significantly lower foveal SCP vascular density than both the PRP and VEDOSS groups (p < 0.001). Additionally, the dcSSc group showed significantly lower foveal SCP vascular density than the PRP group (p < 0.001). However, the perifoveal SCP vascular density values were found to be significantly higher in the dcSSc group compared to the lcSSc group (p < 0.001). Lastly, the FAZ perimeter in the lcSSc group was significantly greater than that in the VEDOSS group (p = 0.017) (**Table 2**).

To summarize, OCT-A provides a non-invasive method for visualizing and quantifying microvascular structures in the retina and choroid. It has demonstrated significant utility in detecting early signs of diseases such as diabetic retinopathy, retinal vascular occlusion, and age-related macular degeneration, offering valuable insights into microvascular changes without the need for invasive dye injections. Additionally, OCT-A has shown promise in assessing microvascular alterations in patients with systemic diseases with evidence suggesting its potential use in evaluating disease activity, early ocular involvement, and medication side effects. Importantly, different associations between OCT-A values and CV-risk factors could be found in patients with ARDs (e.g. negative associations of vessel density with systolic blood pressure, cIMT (98), and age (97)). Moreover, abnormal QRISK3 score and cIMT values were identified as independent risk factors for changes in the retinal vessels (98). These findings suggest that OCT-A may hold diagnostic value in cardiovascular screening and the detection of microangiopathy in ARDs. However, systematic data and consistent longitudinal examinations of its utility in ARDs are still scarce.

### 4.3 Spectral Domain Optical Coherence Tomography (SD-OCT)

#### 4.3.1 Definition of SD-OCT

This method utilizes a spectrometer to simultaneously measure multiple wavelengths of light. This provides faster and more efficient data acquisition of the tissue layers of the retina, while reducing artifacts and improving the resolution of the generated images in comparison to traditional OCT (57, 104). OCT enables a structural examination of the retinal layers and potential diseases. The high axial resolution of 3 to 5 µm allows a precise diagnosis and the possibility to detect early changes in follow-up examinations (105). **Figure 4** shows the different retinal layers at the macula provided by SD-OCT (left section).

#### 4.3.2 Evidence from the general population

A meta-analysis of 12 studies from Salehi et al. (106) included various SD-OCT parameters to measure the choroidal thickness of patients with obesity (634 patients and 569 controls) and reported thinner ocular layers in isolated regions of the retina in patients with higher BMI than in controls (sub-foveal region (standardized mean difference SMD: -0.24, p = 0.05), region 1.0 mm nasal to fovea (SMD: -0.38, p<0.01; region 1.0 mm temporal to fovea (SMD: -0.38, p = 0.05). In another systemic review from the same authors (107) which included 25 studies with 1,632 cases of age-related macular degeneration and 1,445 healthy controls, a significant thinner subfoveal choroidal thickness in obese individuals was also found (SMD, -0.62, p = 0.0077). In different retinal regions like nasal and temporal to the fovea, they also found significant decreased choroid thickness. These abnormalities could impact the visual function.

Furthermore, OCT of the choroid has a high diagnostic value for various ophthalmological entities. The group of pachychoroid spectrum diseases (among others central serous retinopathy, pachychoroid neovasculopathy and polypoidal choroidal vasculopathy) exhibit dilated choroidal vessels, the so-called pachyvessels, and an increased choroidal thickness, which can be detected by OCT. On the other hand, age-related macular degeneration (108), severe diabetic retinopathy (proliferative or diabetic macular edema) (109) and acute anterior ischemic optic neuropathy (110) are associated with a thin choroidal thickness.

Aydin et al. evaluated in a prospective cross-sectional study the SD-OCT parameters central macular thickness (CMT) and choroidal thickness (CT) in 92 patients with CV-risk factors (measured via SCORE) and 21 healthy individuals. CT was significantly lower at the subfoveal location in all study groups (p < 0.05), as well as in the nasal and temporal quadrants of the high CV-risk and coronary arterial disease group (p < 0.05) (111). This indicates that ocular microvascular changes could be used as a promising new biomarker for predicting the occurrence of coronary heart disease in the future.

A further cross-sectional study compared advanced SD-OCT and OCT-A parameters in chronic hypertension, severe hypertensive retinopathy, and healthy controls (112). The hypertensive group (n = 45 eyes) showed significantly lower OCT parameters compared to healthy controls (n = 50 eyes). Notably, a significant correlation was found between OCT-A and SD-OCT parameters in the hypertensive group, but not in controls. OCT-A also revealed reduced vascular and perfusion density and a significantly larger FAZ area in hypertensive patients. These findings suggest that chronic hypertension or past hypertensive episodes impact retinal microcirculation, ultimately affecting retinal thickness.

To summarize, studies of SD-OCT in the general population found correlations with traditional CV risk factors like BMI and hypertension and with the SCORE system.

#### 4.3.3 Evidence in patients with autoimmune rheumatic diseases

##### 4.3.3.1 Systemic lupus erythematosus

In a study examining 46 SLE patients, 32 without (NLR) and 14 with lupus retinopathy (LR), Bao et al. demonstrated that the superficial retinal capillary plexus (SRCP) density was significantly lower in the NLR group than in the 50 healthy control subjects (58). Furthermore, the LR group exhibited a further reduction in density (control 5.8 ± 0.5 vs. NLR 5.6 ± 0.4, p = 0.007; vs. LR 5.3 ± 0.5, p < 0.001). The authors hypothesized that this could be an early indicator of alterations in ocular structures, as well as a potential marker for disease progression (58).

##### 4.3.3.2 Systemic sclerosis

Pieklarz et al. compared in two studies (2023 and 2024) 33 SSc patients to 40 healthy controls via SD-OCT and found that the peripapillary choroidal vascularity index was significantly lower in patients (2023: 64.25 ± 1.94 vs. 65.73 ± 2.12, p < 0.001 2024: 67.26 ± 2.63 vs. 66.30 ± 2.82 p<0,05) (113, 114). The choroidal vascularity index (CVI) calculates the ratio of the luminal area (LA) to the total choroidal area (TCA) and thus enables a quantitative analysis. Their results place a new focus on the impairment of the choroid, which would support the vascular hypothesis for an elevated risk of glaucomatous optic neuropathy in patients with SSc.

CVI may be altered in various ocular and systemic diseases, however, so far it has only been applied in studies, validation in large population-based studies is lacking and the clinical relevance is not conceivable.

In a further study, Carnevali et al. (99) highlighted the ability of SD-OCT to detect ocular microvascular density abnormalities in patients with SSc. They analyzed the eyes of 20 SSc patients and 20 control subjects. Patients had significantly lower deep capillary plexus vessel density compared to control (47.29 (3.49) vs. 50.81 (3.71), p = 0.00). However, no significant difference was observed in CVI and superficial capillary plexus (SCP) (CVI: 66.58 (1.13) vs. 66.70 (1.83), p = 0.80; SCP: 45.67 (5.21) vs. 45.64 (3.22), p = 0.98) (**Table 2**).

To our knowledge, there are no studies examining SD-OCT in RA patients to date.

To summarize, SD-OCT is an advanced imaging technique that allows for high-resolution, non-invasive structural analysis of the retina and choroid. It has shown benefits in assessing ocular changes in various conditions, such as obesity, age-related macular degeneration, and diabetic retinopathy, where it can detect both thinning and thickening of the choroid. The few existing data on SD-OCT also suggest a possible value in identifying early microvascular alterations and potential ocular complications in ARDs like SLE and SSc. However, associations of SD-OCT with CV associated parameters have been only examined in the general population and not in patients with the included ARDs.

### 4.4 Retrobulbar color Doppler

#### 4.4.1 Definition of retrobulbar color Doppler

The retrobulbar color Doppler examination is used to assess ocular blood flow, which is frequently impaired in the presence of CV disease, including carotid artery occlusion, cerebrovascular disease, heart failure, and acute coronary syndrome (61). The method allows for the visualization of the direction and speed of blood flow without providing any information about vessel diameter. Consequently, in some studies, OCT measurements are integrated to obtain comprehensive data on vessel diameter, density and anatomical course (61). The following parameters are determined: the peak systolic flow velocity (PSV), the end diastolic flow velocity (EDV), and the resistance index (RI). The RI is defined as the ratio of the difference between PSV and EDV to PSV (RI = (PSV − EDV)/PSV) (61).

#### 4.4.2 Evidence from the general population

Retrobulbar Doppler indices were evaluated in 66 patients with transient ischemic attack (TIA) or minor stroke (115). Patients with carotid occlusive disease exhibited reduced flow velocities in the OA and CRA (all p < 0.02). In this work, it could be shown that CDI is suitable for the detection of carotid occlusion. Measuring blood flow velocities in the OA and CRA was pointed out as a valid method to identify patients with carotid stenosis, indicating its possible use to give additional information on hemodynamics of carotid arterial disease (115).

Similarly, Reynolds et al. demonstrated that Doppler ultrasound is an effective diagnostic tool for detecting ipsilateral stenosis or occlusion of the ACI. In a study of 152 patients, a reversed flow direction in the orbital artery on one side was found to be a highly specific (100%) marker for high-grade ipsilateral carotid artery stenosis or occlusion (116).

In another study of 18 patients with chronic heart failure symptoms and left ventricle ejection fraction below 55% compared to 21 healthy controls, Almeida-Freitas et al. (117) found that patients revealed significantly lower mean diastolic velocities (5.14 ± 2.4 cm/s vs. 7.44 ± 3.5 cm/s, p = 0.007) and higher RI (0.76 ± 0.08 vs. 0.70 ± 0.08, p=0.04) of the ophthalmic artery (OA). Additionally, they showed a negative correlation between systolic blood pressure and the RI of the OA (r = -0.47, p = 0.007), as well as a positive correlation between diastolic velocity of the OA and systolic blood pressure (r = 0.44, p = 0.02). The authors concluded a possible association to low cardiac output.

Meng et al. summarized data of changes in retrobulbar blood flow measured with retrobulbar color Doppler in a relevant meta-analysis (118). They investigated 13 prospective studies involving 912 eyes from diabetic patients and 553 eyes from healthy controls. The comparison revealed significant differences in color Doppler ultrasound between diabetic eyes without retinopathy and healthy eyes. Specifically, PSV and RI of the ophthalmic artery were elevated (mean difference PSV: 2.25, 95% CI 0.80-3.71, p = 0.002; RI: 0.03, 95% CI 0.01-0.66, p = 0.02). Additionally, both the PSV and EDV of the CRA were decreased (mean difference PSV: -2.44, 95% CI -2.41 to -0.66; EDV: -0.65, 95% CI -1.13 to -0.18; p = 0.07 for both). Furthermore, significant differences were observed between patients with diabetic retinopathy and healthy controls in the following parameters: EDV of the OA and the CRA were significantly reduced in patients (mean difference OA: –1.59, 95% CI –2.46 to –0.72; CRA: –1.18, 95% CI –1.52 to –0.84; both p < 0.001). Moreover, the RI of the OA was higher in the patient cohort compared to the control group (mean difference RI: 0.05, 95% CI 0.02 to 0.08; p = 0.008). These findings suggest that retrobulbar color Doppler could potentially be a valuable diagnostic tool for evaluating microangiopathy in diabetic patients.

Karami et al. conducted a comparative analysis of hemodynamic changes in retrobulbar vessels between diabetic retinopathy patients (n = 98) and a healthy control group (n = 25). They found a significantly higher RI (p = 0.009) and pulsatility index (PI, p = 0.029) in the ophthalmic arteries of diabetic retinopathy patients than in controls (119).

#### 4.4.3 Evidence in patients with autoimmune rheumatic diseases

##### 4.4.3.1 Rheumatoid arthritis

Kal et al. have examined ocular blood flow in patients with RA (60). A total of 20 RA patients and 20 healthy control subjects were included in the study. In each eye, the OA and the CRA were examined with retrobulbar color Doppler ultrasound using a 7.5-MHz linear phase probe. The following parameters were determined: PSV, EDV, and RI. The PSV values of the patients were significantly higher for the OA (p = 0.001; p < 0.001) and the CRA (p = 0.020; p = 0.004). Similarly, the RI values of patients from the ophthalmic (p = 0.001) and CRA (p = 0.005) were significantly higher. In addition, OCT revealed that the perifoveal and subfoveal choroidal thickness were reduced in the patient group.

Unal et al. conducted similar Doppler examinations in a RA and a control group, analyzing 25 patients with active RA and comparing them with 24 healthy subjects (120). A significant positive correlation was observed between the RI of the OA and the Disease Activity Score (DAS 28) (p = 0.02, r = 0.199). Additionally, median RI values of the OA, posterior ciliary artery (PCA), and CRA differed significantly between patients with active RA (evaluated by DAS 28) and the control group (p < 0.05).

These findings are consistent with those of Erdogmus et al., who also utilized Doppler sonography to investigate orbital blood flow in 35 RA patients compared to 35 healthy subjects (121). Significant differences were observed in PSV, EDV, and RI of the ophthalmic artery and OA, as well as RI of the CRA between the control and RA patient groups. Importantly, a slightly lower ocular blood flow was observed in RA patients compared to controls.

##### 4.4.3.2 Systemic lupus erythematosus

Xue et al. demonstrated significant alterations in retrobulbar vessel blood flow in patients with SLE (122). By employing color Doppler ultrasound and evaluating the same arteries and parameters as described above, a correlation was identified between disease activity and the PI values of the ophthalmic arteries. The authors reported decreased blood flow velocity in SLE patients along with increased RI and PI in the PCA and CRA (p < 0.05).

Modrzejewska et al. found retrobulbar resistance disturbances in a study of 43 female SLE patients and a 43 female controls measured by RI in various arteries (123). The color Doppler ultrasound in all measured arteries, the OA, the CRA, the posterior lateral ciliary artery (LPCA) and the medial posterior ciliary artery (MPCA), showed a significant increase in RI in the patient group compared to the controls (mean values; ophthalmic artery 0,73 vs. 0,68; CRA 0,67 vs. 0,64; LPCA 0,67 vs. 0,61; MPCA 0,66 vs 0,60; all p < 0.01). In addition, correlations were identified between age and the CRA-RI of SLE patients (p = 0.0376).

Similarly, Wright et al. (124) utilized Doppler sonography to investigate changes in microangiography in 54 SLE patients and 32 control subjects. They examined the OA, CRA and common carotid artery (CA) for changes in the morphology of the flow velocity waveforms and tested the correlation with RI. No significant difference was observed between the groups with regard to RI in any of the evaluated arteries (OA 0.71 ± 0.08 vs 0.70 ± 0.10; CRA 0.68 ± 0.11 vs 0.70 ± 0.09; CA 0.69 ± 0.10 vs 0.69 ± 0.11). However, the morphology of the flow curves showed different dynamics in the microvasculature of the ocular vessels in the SLE patients. Moreover, a significant difference was observed in the morphology of the velocity waveform within the low frequency range (1.0–1.8 Hz) in SLE patients compared to controls, particularly in the OA and the CRA (p < 0.05) (**Table 2**).

To summarize, retrobulbar color Doppler ultrasound is a valuable diagnostic tool for assessing ocular blood flow in systemic diseases. It allows for measuring the PSV, EDV, RI and PI in key ocular vessels such as the OA and the CRA. Current studies revealed promising insights into autoimmune diseases like RA and SLE by identifying changes in blood flow and vascular resistance in ocular and other vessels. Given that alterations in ultrasound indices in the general population are often linked to increased CV risk or past CV events, it is possible that similar associations exist in rheumatic diseases. However, large longitudinal studies with follow-up are needed to further explore and clarify these associations.

## 5 Discussion

It is well established that patients with ARDs are at an elevated risk of developing CV disease (e.g. stroke, coronary artery occlusion myocardial infarction). Therefore, early detection of CV risk factors and pathological vascular changes is crucial in these patient populations. Nevertheless, current evidence supporting the effective stratification and early detection of CV risk remains limited, and traditional CV risk scores have been shown to underestimate risk in ARDs. Several surrogate markers have been proposed as potential means of identifying high-risk patients in the early stages. However, these markers are not yet used routinely in clinical settings.

Microangiopathy has been described to lead to an increased vascular resistance and therefore reduced blood flow to the end organs. Consequently, the eye, often referred to as the “window to the heart,” appears to be a valuable organ for assessing microvascular changes. Ocular analyses seem to be able to reveal CV risk before other surrogate parameters do. The non-invasiveness of the measurements is particularly favorable. In particular, OCT-A appears to be a highly promising technique, offering a relatively simple and highly reproducible approach. Another advantage is the standardized evaluation of results, which is based on automated software. SD-OCT also enables faster and more efficient data collection of the retina while reducing artifacts compared to conventional OCT. Additionally, RVA offers several advantages, including cost-effectiveness, the absence of radioactive radiation, and robust inter-examiner reproducibility. The advantages of retrobulbar color imaging include its accessibility in numerous clinical settings and its capacity to evaluate vascular conditions and detect other ocular pathologies, which can be readily identified by trained personnel. In this review, we provided a comprehensive overview of these markers, examining their associations with key CV risk factors, disease activity, surrogate CV parameters, and traditional CV risk scores.

The studies discussed herein have however several limitations. First, small sample sizes and heterogeneity in methodologies and measured parameters limit the generalizability of results and prevent the recommendation of a standardized approach for incorporating these markers into CV risk stratification strategies. On the other hand, these markers have demonstrated predictive value for CV events in the general population, and initial data in ARDs show promising potential. Second, the studies did not allow for prognostic conclusions regarding the value of the included CV surrogate markers in predicting actual mortality or morbidity in ARDs, as most were not conducted in a longitudinal manner. Future research should focus on larger patient populations and establish associations between these surrogate markers and hard CV outcomes through well-designed follow-up studies.

In conclusion, this review underscores the opportunities provided by various methods for assessing the eye’s microvascular status in patients with ARDs. We also aimed to highlight the significance of early detection of risk factors and pathological vascular changes in these patient populations. Looking ahead, artificial intelligence holds the potential to significantly enhance the evaluation of ocular imaging, offering a promising approach for the early detection of CV risk. As early microvascular lesions can be reversed, incorporating diagnostic methods such as OCT-A with advanced imaging techniques, RVA, and retrobulbar color Doppler into regular check-ups could facilitate early diagnosis, prevent CV events, and ultimately improve patient outcomes and quality of life.

## Data Availability

The original contributions presented in the study are included in the article/Supplementary Material. Further inquiries can be directed to the corresponding author.

## References

[1] Deane KD El-Gabalawy H . Pathogenesis and prevention of rheumatic disease: focus on preclinical RA and SLE. Nat Rev Rheumatol. (2014) 10:212–28. doi: 10.1038/nrrheum.2014.6 PMC4090326 24514912

[2] Wibetoe G Sexton J Ikdahl E Rollefstad S Kitas GD van Riel P . Prediction of cardiovascular events in rheumatoid arthritis using risk age calculations: evaluation of concordance across risk age models. Arthritis Res Ther. (2020) 22:90. doi: 10.1186/s13075-020-02178-z 32326974 PMC7178602

[3] Lai CH Hsieh CY Barnado A Huang LC Chen SC Tsai LM . Outcomes of acute cardiovascular events in rheumatoid arthritis and systemic lupus erythematosus: a population-based study. Rheumatol (Oxford). (2020) 59:1355–63. doi: 10.1093/rheumatology/kez456 PMC7849999 31600392

[4] Zimba O Gasparyan AY . Cardiovascular issues in rheumatic diseases. Clin Rheumatol. (2023) 42:2535–9. doi: 10.1007/s10067-023-06656-y 37269421

[5] Mal K Kumar R Mansoor F Kaur N Kumar A Memon S . Risk of major adverse cardiovascular events in patients with rheumatoid arthritis. Cureus. (2020) 12:e12246. doi: 10.7759/cureus.12246 33505813 PMC7823064

[6] Triantafyllias K Leiß R Dreher M Schwarting A . Depressive symptoms in early rheumatoid arthritis: Within the rheumatism network ADAPTHERA. Z Rheumatol. (2019) 78:670–6. doi: 10.1007/s00393-019-0596-9 31016369

[7] Schwarting A Möckel T Lütgendorf F Triantafyllias K Grella S Boedecker S . Fatigue in SLE: diagnostic and pathogenic impact of anti-N-methyl-D-aspartate receptor (NMDAR) autoantibodies. Ann Rheum Dis. (2019) 78:1226–34. doi: 10.1136/annrheumdis-2019-215098 31186256

[8] Lu X Wang Y Zhang J Pu D Hu N Luo J . Patients with systemic lupus erythematosus face a high risk of cardiovascular disease: A systematic review and Meta-analysis. Int Immunopharmacol. (2021) 94:107466. doi: 10.1016/j.intimp.2021.107466 33636561

[9] Mackey RH Kuller LH Moreland LW . Update on cardiovascular disease risk in patients with rheumatic diseases. Rheum Dis Clin North Am. (2018) 44:475–87. doi: 10.1016/j.rdc.2018.03.006 30001787

[10] Cen X Feng S Wei S Yan L Sun L . Systemic sclerosis and risk of cardiovascular disease: A PRISMA-compliant systemic review and meta-analysis of cohort studies. Med (Baltimore). (2020) 99:e23009. doi: 10.1097/MD.0000000000023009 PMC7676589 33217802

[11] Fors Nieves CE Izmirly PM . Mortality in systemic lupus erythematosus: an updated review. Curr Rheumatol Rep. (2016) 18:21. doi: 10.1007/s11926-016-0571-2 26984805

[12] Tektonidou MG . Cardiovascular disease risk in antiphospholipid syndrome: Thrombo-inflammation and atherothrombosis. J Autoimmun. (2022) 128:102813. doi: 10.1016/j.jaut.2022.102813 35247655

[13] Houben E Penne EL Voskuyl AE van der Heijden JW Otten RHJ Boers M . Cardiovascular events in anti-neutrophil cytoplasmic antibody-associated vasculitis: a meta-analysis of observational studies. Rheumatol (Oxford). (2018) 57:555–62. doi: 10.1093/rheumatology/kex338 29029294

[14] Restivo V Candiloro S Daidone M Norrito R Cataldi M Minutolo G . Systematic review and meta-analysis of cardiovascular risk in rheumatological disease: Symptomatic and non-symptomatic events in rheumatoid arthritis and systemic lupus erythematosus. Autoimmun Rev. (2022) 21:102925. doi: 10.1016/j.autrev.2021.102925 34454117

[15] Bartels CM Buhr KA Goldberg JW Bell CL Visekruna M Nekkanti S . Mortality and cardiovascular burden of systemic lupus erythematosus in a US population-based cohort. J Rheumatol. (2014) 41:680–7. doi: 10.3899/jrheum.130874 PMC3975689 24532834

[16] Berger M Fesler P Roubille C . Arterial stiffness, the hidden face of cardiovascular risk in autoimmune and chronic inflammatory rheumatic diseases. Autoimmun Rev. (2021) 20:102891. doi: 10.1016/j.autrev.2021.102891 34229047

[17] Ballocca F D’Ascenzo F Moretti C Omedè P Cerrato E Barbero U . Predictors of cardiovascular events in patients with systemic lupus erythematosus (SLE): a systematic review and meta-analysis. Eur J Prev Cardiol. (2015) 22:1435–41. doi: 10.1177/2047487314546826 25139772

[18] Conroy RM Pyörälä K Fitzgerald AP Sans S Menotti A De Backer G . Estimation of ten-year risk of fatal cardiovascular disease in Europe: the SCORE project. Eur Heart J. (2003) 24:987–1003. doi: 10.1016/S0195-668X(03)00114-3 12788299

[19] Assmann G Cullen P Schulte H . Simple scoring scheme for calculating the risk of acute coronary events based on the 10-year follow-up of the prospective cardiovascular Münster (PROCAM) study. Circulation. (2002) 105:310–5. doi: 10.1161/hc0302.102575 11804985

[20] D’Agostino RB Vasan RS Pencina MJ Wolf PA Cobain M Massaro JM . General cardiovascular risk profile for use in primary care: the Framingham Heart Study. Circulation. (2008) 117:743–53. doi: 10.1161/CIRCULATIONAHA.107.699579 18212285

[21] Romanens M Adams A Warmuth W . PROCAM based myocardial infarction risk in relation to global vascular disease risk: observations from the ARCO cohort study. Swiss Med Wkly. (2022) 152:w30111. doi: 10.4414/SMW.2022.w30111 35201684

[22] Schiborn C Kühn T Mühlenbruch K Kuxhaus O Weikert C Fritsche A . A newly developed and externally validated non-clinical score accurately predicts 10-year cardiovascular disease risk in the general adult population. Sci Rep. (2021) 11:19609. doi: 10.1038/s41598-021-99103-4 34608230 PMC8490374

[23] Hageman S Pennells L Ojeda F Kaptoge S Kuulasmaa K de Vries T . SCORE2 risk prediction algorithms: new models to estimate 10-year risk of cardiovascular disease in Europe. Eur Heart J. (2021) 42:2439–54. doi: 10.1093/eurheartj/ehab309 PMC8248998 34120177

[24] De Vries TI Cooney MT Selmer RM Hageman SHJ Pennells LA Wood A . SCORE2-OP risk prediction algorithms: estimating incident cardiovascular event risk in older persons in four geographical risk regions. Eur Heart J. (2021) 42:2455–67. doi: 10.1093/eurheartj/ehab312 PMC8248997 34120185

[25] Agca R Heslinga SC Rollefstad S Heslinga M McInnes IB Peters MJ . EULAR recommendations for cardiovascular disease risk management in patients with rheumatoid arthritis and other forms of inflammatory joint disorders: 2015/2016 update. Ann Rheum Dis. (2017) 76:17–28. doi: 10.1136/annrheumdis-2016-209775 27697765

[26] Crowson CS Gabriel SE Semb AG van Riel P Karpouzas G Dessein PH . Rheumatoid arthritis-specific cardiovascular risk scores are not superior to general risk scores: a validation analysis of patients from seven countries. Rheumatol (Oxford). (2017) 56:1102–10. doi: 10.1093/rheumatology/kex038 PMC5850220 28339992

[27] Assmann G Schulte H Cullen P Seedorf U . Assessing risk of myocardial infarction and stroke: new data from the Prospective Cardiovascular Münster (PROCAM) study. Eur J Clin Invest. (2007) 37:925–32. doi: 10.1111/j.1365-2362.2007.01888.x 18036028

[28] Kannel WB McGee D Gordon T . A general cardiovascular risk profile: the Framingham Study. Am J Cardiol. (1976) 38:46–51. doi: 10.1016/0002-9149(76)90061-8 132862

[29] Wilson PW D’Agostino RB Levy D Belanger AM Silbershatz H Kannel WB . Prediction of coronary heart disease using risk factor categories. Circulation. (1998) 97:1837–47. doi: 10.1161/01.CIR.97.18.1837 9603539

[30] Sivakumaran J Harvey P Omar A Tayer-Shifman O Urowitz MB Gladman DD . Assessment of cardiovascular risk tools as predictors of cardiovascular disease events in systemic lupus erythematosus. Lupus Sci Med. (2021) 8. doi: 10.1136/lupus-2020-000448 PMC8162102 34045359

[31] Barinotti A Radin M Cecchi I Foddai SG Arbrile M Rubini E . Assessing the cardiovascular risk in patients with systemic lupus erythematosus: QRISK and GAPSS scores head-to-head. Int J Cardiol. (2022) 363:185–9. doi: 10.1016/j.ijcard.2022.06.040 35714714

[32] Drosos GC Vedder D Houben E Boekel L Atzeni F Badreh S . EULAR recommendations for cardiovascular risk management in rheumatic and musculoskeletal diseases, including systemic lupus erythematosus and antiphospholipid syndrome. Ann Rheum Dis. (2022) 81:768–79. doi: 10.1136/annrheumdis-2021-221733 35110331

[33] Triantafyllias K Cavagna L Klonowski A Drott U Fiehn C Wendel S . Possible misclassification of cardiovascular risk by SCORE in antisynthetase syndrome: results of the pilot multicenter study RI.CAR.D.A. Rheumatol (Oxford). (2021) 60:1300–12. doi: 10.1093/rheumatology/keaa525 32940712

[34] Vlachopoulos C Aznaouridis K Stefanadis C . Aortic stiffness for cardiovascular risk prediction: just measure it, just do it! J Am Coll Cardiol. (2014) 63:647–9. doi: 10.1016/j.jacc.2013.10.040 24239659

[35] Van Bortel LM Laurent S Boutouyrie P Chowienczyk P Cruickshank JK De Backer T . Expert consensus document on the measurement of aortic stiffness in daily practice using carotid-femoral pulse wave velocity. J Hypertens. (2012) 30:445–8. doi: 10.1097/HJH.0b013e32834fa8b0 22278144

[36] Jain S Khera R Corrales-Medina VF Townsend RR Chirinos JA . Inflammation and arterial stiffness in humans. Atherosclerosis. (2014) 237:381–90. doi: 10.1016/j.atherosclerosis.2014.09.011 25463062

[37] Middeke M . Central hypertension and arterial stiffness. MMW Fortschr Der Medizin. (2012) 154:61–3. doi: 10.1007/s15006-012-1144-6 23045939

[38] Ben-Shlomo Y Spears M Boustred C May M Anderson SG Benjamin EJ . Aortic pulse wave velocity improves cardiovascular event prediction: an individual participant meta-analysis of prospective observational data from 17,635 subjects. J Am Coll Cardiol. (2014) 63:636–46. doi: 10.1016/j.jacc.2013.09.063 PMC4401072 24239664

[39] Triantafyllias K Thiele LE Cavagna L Baraliakos X Bertsias G Schwarting A . Arterial stiffness as a surrogate marker of cardiovascular disease and atherosclerosis in patients with arthritides and connective tissue diseases: A literature review. Diagnostics (Basel). (2023) 13. doi: 10.3390/diagnostics13111870 PMC10252997 37296720

[40] Triantafyllias K Thiele LE Mandel A Cavagna L Baraliakos X Bertsias G . Arterial stiffness as a surrogate marker of cardiovascular disease and atherosclerosis in patients with vasculitides: A literature review. Diagnostics (Basel). (2023) 13. doi: 10.3390/diagnostics13243603 PMC10743173 38132187

[41] Mandel A Schwarting A Cavagna L Triantafyllias K . Novel surrogate markers of cardiovascular risk in the setting of autoimmune rheumatic diseases: current data and implications for the future. Front Med (Lausanne). (2022) 9:820263. doi: 10.3389/fmed.2022.820263 35847825 PMC9279857

[42] Triantafyllias K De Blasi M Hoffmann I Thomaidis T Drees P Schwarting A . The count of tender rather than swollen joints correlates with aortic stiffness in patients with rheumatoid arthritis. Springerplus. (2016) 5:428. doi: 10.1186/s40064-016-2066-z 27104116 PMC4828367

[43] Triantafyllias K Liverakos S Muthuraman M Cavagna L Parodis I Schwarting A . Cardiovascular risk evaluation in psoriatic arthritis by aortic stiffness and the systemic coronary risk evaluation (SCORE): results of the prospective PSOCARD cohort study. Rheumatol Ther. (2024) 11:897–911. doi: 10.1007/s40744-024-00676-z 38819779 PMC11265042

[44] Triantafyllias K de Blasi M Lütgendorf F Cavagna L Stortz M Weinmann-Menke J . High cardiovascular risk in mixed connective tissue disease: evaluation of macrovascular involvement and its predictors by aortic pulse wave velocity. Clin Exp Rheumatol. (2019) 37:994–1002. 30943141

[45] Stortz M Triantafyllias K Schwarting A Weinmann-Menke J . Vascular stiffness: influencing factors on carotid-femoral pulse wave velocity in systemic lupus erythematosus. Clin Exp Rheumatol. (2020) 38:74–81. 30943131

[46] Triantafyllias K Gauch S Bertsias G Boumpas D Hasseli R Cavagna L . Integrating carotid doppler, grey scale ultrasound, and aortic oscillometry to evaluate macroangiopathy in myositides: the MYOCARD cohort. Rheumatol (Oxford). (2024). doi: 10.1093/rheumatology/keae682 39672800

[47] Triantafyllias K Stortz M de Blasi M Leistner C Weinmann-Menke J Schwarting A . Increased aortic stiffness in patients with fibromyalgia: results of a prospective study on carotid-femoral pulse wave velocity. Clin Exp Rheumatol. (2019) 37 Suppl 116:114–5. 29185967

[48] Bordy R Totoson P Prati C Marie C Wendling D Demougeot C . Microvascular endothelial dysfunction in rheumatoid arthritis. Nat Rev Rheumatol. (2018) 14:404–20. doi: 10.1038/s41584-018-0022-8 29855620

[49] Hedar AM Stradner MH Roessler A Goswami N . Autoimmune rheumatic diseases and vascular function: the concept of autoimmune atherosclerosis. J Clin Med. (2021) 10:4427. doi: 10.3390/jcm10194427 34640445 PMC8509415

[50] Szekanecz Z Koch AE . Vascular involvement in rheumatic diseases: ‘vascular rheumatology’. Arthritis Res Ther. (2008) 10:224. doi: 10.1186/ar2515 18947376 PMC2592799

[51] Sena CM Gonçalves L Seiça R . Methods to evaluate vascular function: a crucial approach towards predictive, preventive, and personalised medicine. EPMA J. (2022) 13:209–35. doi: 10.1007/s13167-022-00280-7 PMC9120812 35611340

[52] Hysa E Cutolo CA Gotelli E Paolino S Cimmino MA Pacini G . Ocular microvascular damage in autoimmune rheumatic diseases: The pathophysiological role of the immune system. Autoimmun Rev. (2021) 20:102796. doi: 10.1016/j.autrev.2021.102796 33722750

[53] Lacolley P Regnault V Laurent S . Mechanisms of arterial stiffening: from mechanotransduction to epigenetics. Arterioscler Thromb Vasc Biol. (2020) 40:1055–62. doi: 10.1161/ATVBAHA.119.313129 32075419

[54] Laurent S Boutouyrie P . The structural factor of hypertension: large and small artery alterations. Circ Res. (2015) 116:1007–21. doi: 10.1161/CIRCRESAHA.116.303596 25767286

[55] Hanssen H Streese L Vilser W . Retinal vessel diameters and function in cardiovascular risk and disease. Prog Retin Eye Res. (2022) 91:101095. doi: 10.1016/j.preteyeres.2022.101095 35760749

[56] Lang GE Enders C Werner JU . New possibilities in retinal diagnostics using OCT angiography. Klin Monbl Augenheilkd. (2016) 233:613–21. doi: 10.1055/s-0042-105325 27187882

[57] Aumann S Donner S Fischer J Müller F . Optical coherence tomography (OCT): principle and technical realization. In: Bille JF , editor. High Resolution Imaging in Microscopy and Ophthalmology: New Frontiers in Biomedical Optics. Springer International Publishing, Cham (2019). p. 59–85. 32091846

[58] Bao L Zhou R Wu Y Wang J Shen M Lu F . Unique changes in the retinal microvasculature reveal subclinical retinal impairment in patients with systemic lupus erythematosus. Microvasc Res. (2020) 129:103957. doi: 10.1016/j.mvr.2019.103957 31733303

[59] Tranquart F Bergès O Koskas P Arsene S Rossazza C Pisella PJ . Color Doppler imaging of orbital vessels: personal experience and literature review. J Clin Ultrasound. (2003) 31:258–73. doi: 10.1002/jcu.10169 12767021

[60] Kal A Duman E Sezenöz AS Ulusoy MO Kal Ö . Evaluation of retrobulbar blood flow and choroidal thickness in patients with rheumatoid arthritis. Int Ophthalmol. (2018) 38:1825–31. doi: 10.1007/s10792-017-0656-6 28730400

[61] Böhm EW Grauhan NF Pfeiffer N Gericke A . Measurement of retrobulbar blood flow and vascular reactivity-relevance for ocular and cardiovascular diseases. Diagnostics (Basel). (2023) 13:4–5. doi: 10.3390/diagnostics13233514 PMC10706662 38066755

[62] Hanssen H Vilser W . Retinal vessel analysis - a new method of diagnostics and risk prediction. 1. Bremen: UNI-MED Verlag AG (2019).

[63] Horton WB Barrett EJ . Microvascular dysfunction in diabetes mellitus and cardiometabolic disease. Endocr Rev. (2021) 42:29–55. doi: 10.1210/endrev/bnaa025 33125468 PMC7846151

[64] Koller A Toth P . Contribution of flow-dependent vasomotor mechanisms to the autoregulation of cerebral blood flow. J Vasc Res. (2012) 49:375–89. doi: 10.1159/000338747 PMC3586555 22739136

[65] Guven G Hilty MP Ince C . Microcirculation: physiology, pathophysiology, and clinical application. Blood Purif. (2020) 49:143–50. doi: 10.1159/000503775 PMC7114900 31851980

[66] Lambova SN . Microangiopathy in rheumatic diseases. Life (Basel). (2023) 13. doi: 10.3390/life13020491 PMC9965541 36836847

[67] Brunner H Cockcroft JR Deanfield J Donald A Ferrannini E Halcox J . Endothelial function and dysfunction. Part II: Association with cardiovascular risk factors and diseases. A statement by the Working Group on Endothelins and Endothelial Factors of the European Society of Hypertension. J Hypertens. (2005) 23:233–46. doi: 10.1097/00004872-200502000-00001 15662207

[68] Klein A Molad Y . Hematological manifestations among patients with rheumatic diseases. Acta Haematol. (2021) 144:403–12. doi: 10.1159/000511759 33221805

[69] Farrah TE Dhillon B Keane PA Webb DJ Dhaun N . The eye, the kidney, and cardiovascular disease: old concepts, better tools, and new horizons. Kidney Int. (2020) 98:323–42. doi: 10.1016/j.kint.2020.01.039 PMC7397518 32471642

[70] England BR Thiele GM Anderson DR Mikuls TR . Increased cardiovascular risk in rheumatoid arthritis: mechanisms and implications. Bmj. (2018) 361:k1036. doi: 10.1136/bmj.k1036 29685876 PMC6889899

[71] Patton N Aslam T Macgillivray T Pattie A Deary IJ Dhillon B . Retinal vascular image analysis as a potential screening tool for cerebrovascular disease: a rationale based on homology between cerebral and retinal microvasculatures. J Anat. (2005) 206:319–48. doi: 10.1111/j.1469-7580.2005.00395.x PMC1571489 15817102

[72] Palkovits S Lasta M Told R Schmidl D Boltz A Napora KJ . Retinal oxygen metabolism during normoxia and hyperoxia in healthy subjects. Invest Ophthalmol Visual Sci. (2014) 55:4707–13. doi: 10.1167/iovs.14-14593 25015353

[73] Madamanchi NR Vendrov A Runge MS . Oxidative stress and vascular disease. Arterioscler Thromb Vasc Biol. (2005) 25:29–38. doi: 10.1161/01.ATV.0000150649.39934.13 15539615

[74] Cheung CY Zheng Y Hsu W Lee ML Lau QP Mitchell P . Retinal vascular tortuosity, blood pressure, and cardiovascular risk factors. Ophthalmology. (2011) 118:812–8. doi: 10.1016/j.ophtha.2010.08.045 21146228

[75] Wieberdink RG Ikram MK Koudstaal PJ Hofman A Vingerling JR Breteler MM . Retinal vascular calibers and the risk of intracerebral hemorrhage and cerebral infarction: the Rotterdam Study. Stroke. (2010) 41:2757–61. doi: 10.1161/STROKEAHA.110.599084 21030694

[76] Wang JJ Liew G Klein R RochtChina E Knudtson MD Klein BE . Retinal vessel diameter and cardiovascular mortality: pooled data analysis from two older populations. Eur Heart J. (2007) 28:1984–92. doi: 10.1093/eurheartj/ehm221 17626032

[77] Seidelmann SB Claggett B Bravo PE Gupta A Farhad H Klein BE . Retinal vessel calibers in predicting long-term cardiovascular outcomes: the atherosclerosis risk in communities study. Circulation. (2016) 134:1328–38. doi: 10.1161/CIRCULATIONAHA.116.023425 PMC5219936 27682886

[78] Al-Fiadh AH Wong TY Kawasaki R Clark DJ Patel SK Freeman M . Usefulness of retinal microvascular endothelial dysfunction as a predictor of coronary artery disease. Am J Cardiol. (2015) 115:609–13. doi: 10.1016/j.amjcard.2014.12.011 25591896

[79] Cabrera DeBuc D Somfai GM Koller A . Retinal microvascular network alterations: potential biomarkers of cerebrovascular and neural diseases. Am J Physiol Heart Circ Physiol. (2017) 312:H201–h12. doi: 10.1152/ajpheart.00201.2016 PMC5336575 27923786

[80] Hubbard LD Brothers RJ King WN Clegg LX Klein R Cooper LS . Methods for evaluation of retinal microvascular abnormalities associated with hypertension/sclerosis in the Atherosclerosis Risk in Communities Study. Ophthalmology. (1999) 106:2269–80. doi: 10.1016/S0161-6420(99)90525-0 10599656

[81] Parr JC Spears GFS . General caliber of the retinal arteries expressed as the equivalent width of the central retinal artery. Am J Ophthalmol. (1974) 77:472–7. doi: 10.1016/0002-9394(74)90457-7 4819451

[82] Drobnjak D Munch IC Glümer C Faerch K Kessel L Larsen M . Retinal vessel diameters and their relationship with cardiovascular risk and all-cause mortality in the inter99 eye study: A 15-year follow-up. J Ophthalmol. (2016) 2016:6138659. doi: 10.1155/2016/6138659 28053777 PMC5174182

[83] Wong TY Islam FM Klein R Klein BE Cotch MF Castro C . Retinal vascular caliber, cardiovascular risk factors, and inflammation: the multi-ethnic study of atherosclerosis (MESA). Invest Ophthalmol Vis Sci. (2006) 47:2341–50. doi: 10.1167/iovs.05-1539 PMC2258139 16723443

[84] Kawasaki R Cheung N Wang JJ Klein R Klein BE Cotch MF . Retinal vessel diameters and risk of hypertension: the Multiethnic Study of Atherosclerosis. J Hypertens. (2009) 27:2386–93. doi: 10.1097/HJH.0b013e3283310f7e PMC2935621 19680136

[85] Lee JH Kim SS Kim GT . Microvascular findings in patients with systemic lupus erythematosus assessed by fundus photography with fluorescein angiography. Clin Exp Rheumatol. (2013) 31:871–6. 24021339

[86] Babaoğlu H Baytaroğlu A Torğutalp M Erden A Kadayıfçılar S Kalyoncu U . Abnormal retinal microvasculature found in active rheumatoid arthritis:a different perspective of microvascular health. Turk J Med Sci. (2019) 49:20–6. doi: 10.3906/sag-1806-1 PMC7350799 30761837

[87] Liu M Lovern C Lycett K He M Wake M Wong TY . The association between markers of inflammation and retinal microvascular parameters: A systematic review and meta-analysis. Atherosclerosis. (2021) 336:12–22. doi: 10.1016/j.atherosclerosis.2021.09.025 34607278

[88] Anyfanti P Triantafyllou A Gkaliagkousi E Koletsos N Athanasopoulos G Zabulis X . Retinal vessel morphology in rheumatoid arthritis: Association with systemic inflammation, subclinical atherosclerosis, and cardiovascular risk. Microcirculation. (2017) 24. doi: 10.1111/micc.2017.24.issue-8 28926162

[89] Deiseroth A Marcin T Berger C Infanger D Schäfer J Bannert B . Retinal vessel diameters and physical activity in patients with mild to moderate rheumatic disease without cardiovascular comorbidities. Front Physiol. (2018) 9:176. doi: 10.3389/fphys.2018.00176 29593551 PMC5854847

[90] Sun C Ladores C Hong J Nguyen DQ Chua J Ting D . Systemic hypertension associated retinal microvascular changes can be detected with optical coherence tomography angiography. Sci Rep. (2020) 10:9580. doi: 10.1038/s41598-020-66736-w 32533105 PMC7293289

[91] Tan W Yao X Le TT Tan ACS Cheung CY Chin CWL . The application of optical coherence tomography angiography in systemic hypertension: A meta-analysis. Front Med (Lausanne). (2021) 8:778330. doi: 10.3389/fmed.2021.778330 34859021 PMC8630630

[92] Lee HY Chen J Ying P Xu SH Kang M Zou J . Investigation of altered retinal microvasculature in female patients with rheumatoid arthritis: optical coherence tomography angiography detection. Biosci Rep. (2023) 43:5–6. doi: 10.1042/BSR20230045 PMC10578346 37665319

[93] Ayar K Can ME Koca N Çelik D . Evaluation of retinal vascularization by optical coherence tomography angiography (OCTA) in rheumatoid arthritis, and its relationship with disease activity. Mod Rheumatol. (2021) 31:817–26. doi: 10.1080/14397595.2020.1830740 32997565

[94] Dammacco R Guerriero S Alessio G Dammacco F . Natural and iatrogenic ocular manifestations of rheumatoid arthritis: a systematic review. Int Ophthalmol. (2022) 42:689–711. doi: 10.1007/s10792-021-02058-8 34802085 PMC8882568

[95] Liu LQ Shi WQ Chen J Li QJ Qian L Wei H . Retinal alterations in evaluation of rheumatoid arthritis with chloroquine treatment: A new approach. J Biophotonics. (2023) 16:e202300133. doi: 10.1002/jbio.202300133 37369631

[96] Arfeen SA Bahgat N Adel N Eissa M Khafagy MM . Assessment of superficial and deep retinal vessel density in systemic lupus erythematosus patients using optical coherence tomography angiography. Graefes Arch Clin Exp Ophthalmol. (2020) 258:1261–8. doi: 10.1007/s00417-020-04626-7 32162113

[97] Conigliaro P Cesareo M Chimenti MS Triggianese P Canofari C Aloe G . Evaluation of retinal microvascular density in patients affected by systemic lupus erythematosus: an optical coherence tomography angiography study. Ann Rheum Dis. (2019) 78:287–9. doi: 10.1136/annrheumdis-2018-214235 30242032

[98] Ferrigno S Conigliaro P Rizza S Longo S Nesi C Carlucci F . Relationship between retinal microvascular impairment and subclinical atherosclerosis in SLE. Lupus Sci Med. (2023) 10. doi: 10.1136/lupus-2023-000977 PMC10603324 37852671

[99] Carnevali A Giannaccare G Gatti V Battaglia C Randazzo G Yu AC . Retinal microcirculation abnormalities in patients with systemic sclerosis: an explorative optical coherence tomography angiography study. Rheumatol (Oxford). (2021) 60:5827–32. doi: 10.1093/rheumatology/keab258 33715001

[100] Kılınç Hekimsoy H Şekeroğlu MA Koçer AM Akdoğan A . Analysis of retinal and choroidal microvasculature in systemic sclerosis: an optical coherence tomography angiography study. Eye (Lond). (2020) 34:763–70. doi: 10.1038/s41433-019-0591-z PMC7093531 31554941

[101] Mihailovic N Lahme L Braasch S Rosenberger F Eter N Ehrchen J . Altered ocular microvasculature in patients with systemic sclerosis and very early disease of systemic sclerosis using optical coherence tomography angiography. Sci Rep. (2022) 12:10990. doi: 10.1038/s41598-022-14377-6 35768479 PMC9243093

[102] Cutolo CA Cere A Toma P Cannavacciuolo T Toma C Balito S . Peripheral and ocular microvascular alterations in systemic sclerosis: observations from capillaroscopic assessments, perfusion peripheral analysis, and optical coherence tomography angiography. Rheumatol Int. (2024) 44:107–18. doi: 10.1007/s00296-023-05495-z PMC10766778 37978075

[103] Erturk A Erogul O Kasikci M . Optical coherence tomography angiography is a useful tool for distinguishing primary raynaud’s phenomenon from systemic sclerosis and/or very early disease of systemic sclerosis. Diagnostics (Basel). (2023) 13. doi: 10.3390/diagnostics13152607 PMC10417700 37568970

[104] Fujimoto J Swanson E . The development, commercialization, and impact of optical coherence tomography. Invest Ophthalmol Visual Sci. (2016) 57:OCT1–OCT13. doi: 10.1167/iovs.16-19963 27409459 PMC4968928

[105] Aref AA Conner I Cretara EAZ Downes RA El-Dairi MA Freedman SF . Optical Coherence Tomography in Glaucoma. New York: Thieme Medical Publishers, Inc (2022). Available at: http://www.thieme-connect.de/products/ebooks/book/10.1055/b000000397 (Accessed May 5, 2024).

[106] Salehi MA Karimi A Mohammadi S Arevalo JF . Spectral-domain OCT measurements in obesity: A systematic review and meta-analysis. PloS One. (2022) 17:e0267495. doi: 10.1371/journal.pone.0267495 35476846 PMC9045631

[107] Salehi MA Mohammadi S Gouravani M Rezagholi F Arevalo JF . Retinal and choroidal changes in AMD: A systematic review and meta-analysis of spectral-domain optical coherence tomography studies. Surv Ophthalmol. (2023) 68:54–66. doi: 10.1016/j.survophthal.2022.07.006 35908660

[108] Kim SW Oh J Kwon SS Yoo J Huh K . Comparison of choroidal thickness among patients with healthy eyes, early age-related maculopathy, neovascular age-related macular degeneration, central serous chorioretinopathy, and polypoidal choroidal vasculopathy. Retina. (2011) 31:1904–11. doi: 10.1097/IAE.0b013e31821801c5 21878855

[109] Regatieri CV Branchini L Carmody J Fujimoto JG Duker JS . Choroidal thickness in patients with diabetic retinopathy analyzed by spectral-domain optical coherence tomography. Retina. (2012) 32:563–8. doi: 10.1097/IAE.0B013E31822F5678 PMC3393081 22374157

[110] Schuster AK Steinmetz P Forster TM Schlichtenbrede FC Harder BC Jonas JB . Choroidal thickness in nonarteritic anterior ischemic optic neuropathy. Am J Ophthalmol. (2014) 158:1342–7:e1. doi: 10.1016/j.ajo.2014.09.008 25217855

[111] Aydin E Kazanci L Balikoglu Yilmaz M Akyildiz Akcay F Bayata S . Analysis of central macular thickness and choroidal thickness changes in patients with cardiovascular risk factors. Eye (Lond). (2020) 34:2068–75. doi: 10.1038/s41433-020-0775-6 PMC7784908 31992862

[112] Lee WH Park JH Won Y Lee MW Shin YI Jo YJ . Retinal microvascular change in hypertension as measured by optical coherence tomography angiography. Sci Rep. (2019) 9:156. doi: 10.1038/s41598-018-36474-1 30655557 PMC6336859

[113] Pieklarz B Gińdzieńska-Sieśkiewicz E Zawadzka I Bagrowska M Daniluk J Sidorczuk P . Peripapillary choroidal vascularity index and thickness in patients with systemic sclerosis. Front Med (Lausanne). (2023) 10:1273438. doi: 10.3389/fmed.2023.1273438 37915331 PMC10617027

[114] Pieklarz B Gińdzieńska-Sieśkiewicz E Zawadzka I Bagrowska M Daniluk J Palewski M . Macular choroidal thickness, volume, and vascularity index in patients with systemic sclerosis. Graefes Arch Clin Exp Ophthalmol. (2024) 262:1475–87. doi: 10.1007/s00417-023-06342-4 PMC11031445 38133798

[115] Hu HH Sheng WY Yen MY Lai ST Teng MM . Color Doppler imaging of orbital arteries for detection of carotid occlusive disease. Stroke. (1993) 24:1196–203. doi: 10.1161/01.STR.24.8.1196 8342197

[116] Reynolds PS Greenberg JP Lien LM Meads DC Myers LG Tegeler CH . Ophthalmic artery flow direction on color flow duplex imaging is highly specific for severe carotid stenosis. J Neuroimaging. (2002) 12:5–8. doi: 10.1111/j.1552-6569.2002.tb00082.x 11826597

[117] Almeida-Freitas DB Meira-Freitas D Melo LA Jr. Paranhos A Jr. Iared W Ajzen S . Color Doppler imaging of the ophthalmic artery in patients with chronic heart failure. Arq Bras Oftalmol. (2011) 74:326–9. doi: 10.1590/S0004-27492011000500003 22183990

[118] Meng N Liu J Zhang Y Ma J Li H Qu Y . Color doppler imaging analysis of retrobulbar blood flow velocities in diabetic patients without or with retinopathy: A meta-analysis. J Ultrasound Med. (2014) 33:1381–9. doi: 10.7863/ultra.33.8.1381 25063403

[119] Karami M Janghorbani M Dehghani A Khaksar K Kaviani A . Orbital Doppler evaluation of blood flow velocities in patients with diabetic retinopathy. Rev Diabetes Stud. (2012) 9:104–11. doi: 10.1900/RDS.2012.9.104 PMC3700023 23403706

[120] Unal O Can ME Ozcan A Ozcan ME Erten S Cagil N . Color Doppler imaging of ocular hemodynamic changes in patients with rheumatoid arthritis unrelated to disease activity. Rheumatol Int. (2019) 39:1001–6. doi: 10.1007/s00296-019-04275-y 30864110

[121] Erdogmus B Yazici S Yazici B Ataoglu S Buyukkaya R Yuksel H . Orbital blood flow velocities in patients with rheumatoid arthritis. J Clin Ultrasound. (2007) 35:367–71. doi: 10.1002/jcu.20348 17471579

[122] Xue K Guo T Lei B Chen S Huang L Zhou M . Retrobulbar blood flow velocity in systemic lupus erythematosus assessed by color Doppler imaging. Lupus. (2022) 31:582–7. doi: 10.1177/09612033221088181 35343283

[123] Modrzejewska M Ostanek L Bobrowska-Snarska D Karczewicz D Wilk G Brzosko M . Ocular circulation in systemic lupus erythematosus. Med Sci Monit. (2009) 15:Cr573–8. 19865056

[124] Wright SA O’Prey FM Hamilton PK Lockhart CJ McCann A McHenry MT . Colour Doppler ultrasound of the ocular circulation in patients with systemic lupus erythematosus identifies altered microcirculatory haemodynamics. Lupus. (2009) 18:950–7. doi: 10.1177/0961203309104865 19762395

[125] Page MJ McKenzie JE Bossuyt PM Boutron I Hoffmann TC Mulrow CD . The PRISMA 2020 statement: an updated guideline for reporting systematic reviews. BMJ. (2021) 372:n71. doi: 10.1186/s13643-021-01626-4 33782057 PMC8005924

